# Prion-like domains drive CIZ1 assembly formation at the inactive X chromosome

**DOI:** 10.1083/jcb.202103185

**Published:** 2022-03-15

**Authors:** Sajad Sofi, Louisa Williamson, Gabrielle L. Turvey, Charlotte Scoynes, Claire Hirst, Jonathan Godwin, Neil Brockdorff, Justin Ainscough, Dawn Coverley

**Affiliations:** 1 Department of Biology, University of York, York, UK; 2 York Biomedical Research Institute, University of York, York, UK; 3 College of Science and Engineering, University of Edinburgh, Edinburgh, UK; 4 Department of Biochemistry, University of Oxford, Oxford, UK

## Abstract

CIZ1 forms large assemblies at the inactive X chromosome (Xi) in female fibroblasts in an *Xist* lncRNA-dependent manner and is required for accurate maintenance of polycomb targets genome-wide. Here we address requirements for assembly formation and show that CIZ1 undergoes two direct interactions with *Xist*, via independent N- and C-terminal domains. Interaction with *Xist*, assembly at Xi, and complexity of self-assemblies formed in vitro are modulated by two alternatively spliced glutamine-rich prion-like domains (PLD1 and 2). PLD2 is dispensable for accumulation at existing CIZ1–Xi assemblies in wild-type cells but is required in CIZ1-null cells where targeting, assembly, and enrichment for H3K27me3 and H2AK119ub occur de novo. In contrast, PLD1 is required for both de novo assembly and accumulation at preexisting assemblies and, in vitro, drives formation of a stable fibrillar network. Together they impart affinity for RNA and a complex relationship with repeat E of *Xist*. These data show that alternative splicing of two PLDs modulates CIZ1’s ability to build large RNA–protein assemblies.

## Teaser

This paper dissects the requirement for glutamine-rich prion-like domains in the formation of intranuclear CIZ1 protein assemblies at the inactive X chromosome. It highlights the influence of alternative splicing and implicates a polyglutamine domain in maintenance of the epigenetic state.

## Introduction

X-chromosome inactivation (XCI) is initiated by the long noncoding RNA (lncRNA) product of the X-linked gene *Xist* (X-inactive specific transcript) in the blastocyst of developing females (Brockdorff et al., 1992; Brown et al., 1992), leading to equalization of X-linked gene dosage between males and females (Penny et al., 1996). Once established gene silencing is maintained through subsequent cell generations, defining distinct initiation and maintenance phases of XCI (Wutz and Jaenisch, 2000). Initiation can be modeled in differentiating embryonic stem cells, where recruitment of CIP1-interacting zinc finger protein 1 (CIZ1) to the inactive X chromosome (Xi) is dependent on the repeat E region of *Xist* (Ridings-Figueroa et al., 2017; Sunwoo et al., 2017). Although this occurs concurrently with expression of *Xist* and with establishment of Xi chromatin, CIZ1 is not essential for establishment of XCI, and mice lacking CIZ1 develop normally (Ridings-Figueroa et al., 2017). CIZ1 becomes functionally relevant later, during maintenance of XCI, which we study here using differentiated primary embryonic fibroblasts (PEFs). In these cells, retention of *Xist* at Xi and maintenance of repressive chromatin modifications H2AK119ub and H3K27me3 (deposited by polycomb repressive complex 1 [PRC1] and PRC2, respectively) are dependent on CIZ1. At this stage CIZ1 forms large assemblies at Xi in female cells (Ridings-Figueroa et al., 2017) as well as much smaller nucleus-wide foci in both sexes (Ainscough et al., 2007).

Deletion of CIZ1 has revealed a role in high-fidelity maintenance of PRC 1/2 gene sets that is linked with a replication-coupled process of chromatin relocation (Stewart et al., 2019). At this point in the cell cycle, CIZ1–Xi assemblies undergo a shift in properties that alter their relationship with RNA. This makes the formation and cell cycle–dependent stabilization of CIZ1–Xi assemblies of particular interest for what they may be able to reveal about the stability and fluidity of RNA-dependent subnuclear assemblies in general. Notably, loss of CIZ1 affects expression of ∼2% of genes, both X-linked and elsewhere in the genome (Ridings-Figueroa et al., 2017), suggesting that the mechanism by which it contributes to preservation of epigenetic landscape at Xi may be applicable to other CIZ1 foci and other loci.

It was recently hypothesized that *Xist*-dependent protein assemblies are phase-separated condensates that form a membrane-less compartment in the vicinity of Xi (Cerase et al., 2019; Pandya-Jones et al., 2020). Membrane-less compartments, such as Cajal bodies and nuclear speckles, are micron-sized assemblies of proteins or RNA–protein complexes formed by liquid–liquid phase separation (LLPS; Shin and Brangwynne, 2017). Most are sphere-like, but others, such as the TIS (TIS11B-RNA) granule network, form mesh-like structures (Ma et al., 2020 *Preprint*). In most cases, LLPS involves RNA-binding proteins (RBPs) harboring prion-like domains (PLDs). PLDs are intrinsically disordered regions with low sequence complexity that contain repeats of polar amino acids such as polyglutamine (polyQ) that favor weak protein–protein interactions (Maharana et al., 2018). They play pivotal roles in normal cell physiology, however sometimes their physiological state is perturbed leading to abnormal protein aggregation or maturation to amyloid-like fibers associated with disease (Da Cruz and Cleveland, 2011).

Here we address the requirements for CIZ1 assembly at Xi in differentiated cells and implicate two alternatively spliced PLD domains. Both contribute to de novo formation of functional CIZ1 assemblies at Xi, accompanied by repressive chromatin modifications. The data support the idea that these assemblies are localized at Xi by direct interaction with *Xist* via at least two independent CIZ1 interaction interfaces, one with preference for *Xist* repeat E.

## Results

### Alternatively spliced PLDs modulate CIZ1 assembly at Xi

**Figure 1.**
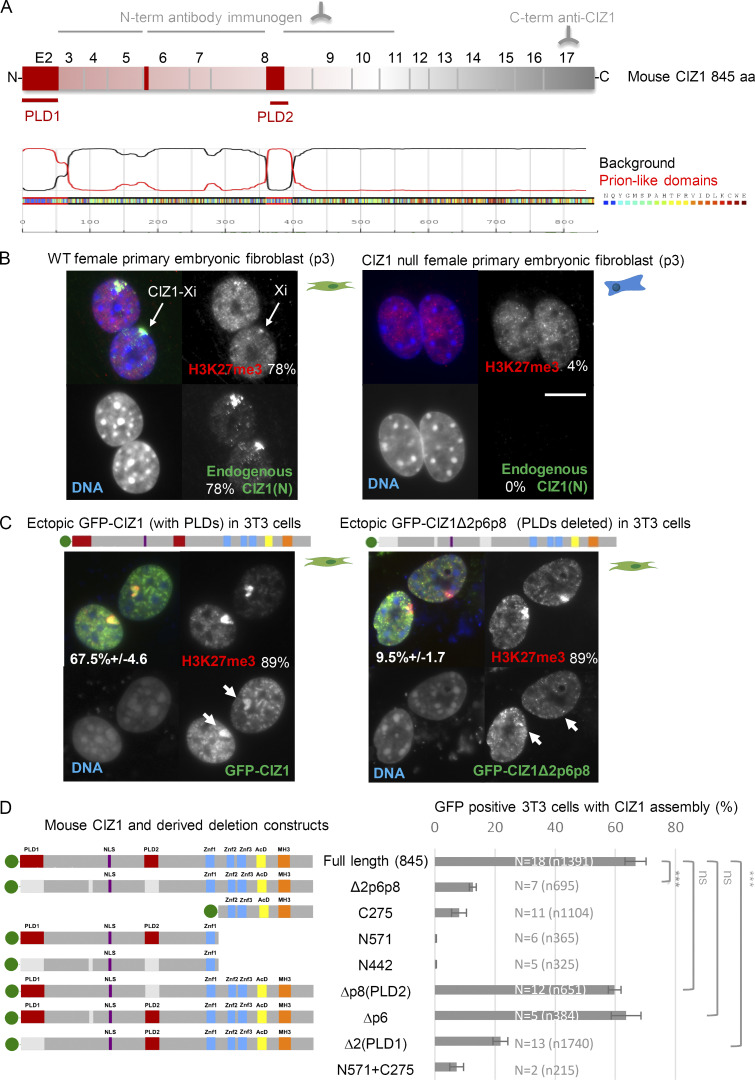
**Alternative splicing of CIZ1 PLDs regulates assembly at Xi. (A)** Schematic showing translated exons 2–17, giving rise to predicted full-length murine CIZ1, specified by RefSeq accession no. NP_082688.1. Alternatively spliced exon 2 excluded from CIZ1Δ2p6p8, as well as partially excluded exons 6 and 8, are indicated in red. The location of immunogens for anti-CIZ1 N-terminal domain antibody and anti-CIZ1 C-terminal domain antibody are shown above. Below, PLDs identified by PLAAC (Lancaster et al., 2014) align with alternatively spliced exons 2 (PLD1) and 8 (PLD2). **(B)** Example images of murine PEFs (p3) derived from WT and CIZ1-null mice (Ridings-Figueroa et al., 2017), showing endogenous CIZ1 detected with anti-CIZ1(N) antibody (green), H3K27me3 (red), and DNA (blue) in merged images. Bar is 10 μm. In this WT population of cycling cells, 78% had colocalized CIZ1/H3K27me3-marked Xi's, compared with 4% marked only with H3K27me3 in CIZ1-null cells (0% CIZ1). **(C)** Expression of ectopic GFP-CIZ1 or alternatively spliced variant GFP-CIZ1Δ2p6p8 (Coverley et al., 2005), 24 h after transient transfection into WT cells (endogenous H3K27me3-Xi frequency 88.9% ± 2.4%, *N* = 5, *n* = 756). Arrows show accumulation of CIZ1, but not CIZ1Δ2p6p8, at sites of H3K27me3-enriched chromatin. The frequency with which CIZ1 assemblies are observed at Xi is indicated, with SEM. **(D)** Left: Illustration of CIZ1 deletion and truncation constructs missing combinations of exons 2, 6, and 8 (shown in red where present or in gray if deleted). Green circles, GFP. Right: Their ability to assemble at Xi in cycling murine 3T3 cells, 24 h after transfection, with SEM, and comparisons between key constructs by Student’s two tailed *t* test. Example images for all constructs are given in Fig. S1 C. *N* indicates the number of repeat experiments and *n* the number of nuclei scored (full-length GFP-CIZ1845, *N* = 18, *n* = 1,391; CIZ1Δ2p6p8, *N* = 7, *n* = 695; C275, *N* = 11, *n* = 1,104; N571, *N* = 6, *n* = 365; N442, *N* = 5, *n* = 325; Δp8(PLD2), *N* = 12, *n* = 651; Δp6, *N* = 5, *n* = 384; Δ2(PLD1), *N* = 13, *n* = 1,740; N571+C275, *N* = 2, *n* = 215).

**Figure S1.**
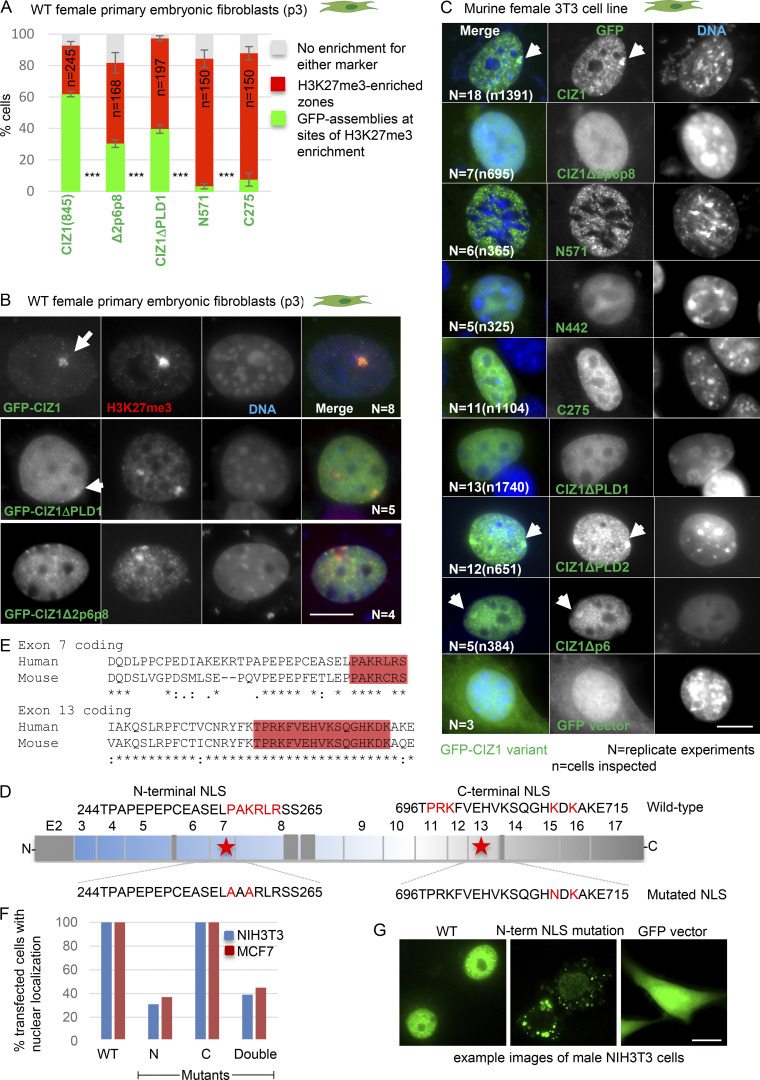
**Sequence requirements for nuclear localization; accumulation of GFP-CIZ1 at Xi.** (Related to Fig. 1.) **(A)** Quantification of the ability of the indicated constructs to form assemblies 24 h after transient transfection into WT PEFs (p3), shown relative to H3K27me3-marked Xi's, which are evident in typically 80–90% of individual populations of recipient cells. Results show mean frequencies from at least four experiments for CIZ1, CIZ1Δ2p6p8, and CIZ1ΔPLD1 (*N* values on images in B) and one experiment with three replicate transfections for N571 and C275. Results are shown as mean % with SEM. Significance indicators, calculated by Student’s two tailed *t* test, compare deletion constructs to full length CIZ1. *n* = 245/168/197/150/150 for CIZ1(845), CIZ1Δ2p6p8, CIZ1ΔPLD1, N571, C275, respectively. **(B)** Example images, acquired under standardized imaging conditions, showing H3K27me3-marked Xi's (red) and GFP-CIZ1 assemblies (green), which, for the variant forms, not only are reduced in frequency, but when present are typically smaller, more diffuse entities (example nucleus with weak assembly is shown for CIZ1ΔPLD1. Values in A include weak assemblies. **(C)** Representative images (related to Fig. 1 D, which gives mean percentage values ± SEM) showing the behavior of the indicated GFP-CIZ1 variants (green) 24 h after transient transfection into 3T3 cells. DNA was counterstained with DAPI (blue) during the wash steps, except CIZ1Δp6, which was mounted in Vectashield with DAPI. Bar is 10 μm. Arrows indicate Xi's with accumulated GFP. All constructs, except GFP empty vector, are exclusively nuclear and resistant to prefixation detergent exposure. Image capture parameters were not standardized across the different constructs. **(D)** Exon map of human CIZ1 showing location of two putative NLSs (starred) detected by PSORTII, a classic NLS in the replication domain of CIZ1 (N) encoded by exon 7, and a bipartite NLS (Robbins et al., 1991) in the anchor domain of CIZ1 (C), encoded by exon 13. Sequences encompassing the predicted NLS (amino acid positions from human protein reference sequence BAA85783.1), with key amino acids in red. Below, products of mutagenesis with changed amino acids in red. **(E)** Alignments showing human and mouse CIZ1 exons 7 and 13, with conserved NLSs highlighted in red. **(F)** Proportion of transfected cells in which nuclear GFP fluorescence exceeds cytoplasmic fluorescence, for the indicated four constructs, 24 h after transient transfection into male NIH3T3 cells (mouse) or female MCF7 cells (human). N and C, individually mutated NLSs; double, mutation of both in the same construct. **(G)** Representative images showing GFP-CIZ1 (green) without and with N-terminal NLS mutation, in NIH3T3 cells. Bar is 10 μm. ***, P < 0.001.

Mouse CIZ1 (Fig. 1 A) encodes two functionally distinct and partially characterized regions that we previously referred to as N-terminal replication domain (within amino acids 1–536 of RefSeq accession no. NP_082688.1), which promotes cyclin-dependent initiation of DNA replication in vitro (Copeland et al., 2015), and C-terminal nuclear matrix anchor domain (within 537–845), which supports association with non-chromatin nuclear structures (Ainscough et al., 2007). Antibodies directed against epitopes in either the N- or C-terminal regions detect large assemblies of endogenous CIZ1 in the location of Xi in WT female fibroblasts (Fig. 1 B), activated lymphocytes, and differentiated embryonic stem cells (Ridings-Figueroa et al., 2017; Sunwoo et al., 2017), but not in cells derived from CIZ1-null mice (Fig. 1 B). Accumulation of CIZ1 protein assemblies at Xi can be modeled in PEFs when ectopic murine full-length GFP-CIZ1 is expressed from an integrated inducible vector (Ridings-Figueroa et al., 2017) or by transient transfection into WT cells (Fig. 1 C). After transient transfection, GFP-CIZ1 assemblies form at Xi's, identified by co-staining for H3K27me3, with variable efficiency depending on cell type; evident in 67% of cycling female 3T3 cells after 24 h (Fig. 1 C), and 62% of WT female PEFs (p3; Fig. S1, A and B).

In contrast, a naturally occurring, alternatively spliced variant of murine CIZ1 cloned from an embryonic day 11 cDNA library (previously termed embryonic CIZ1 or ECIZ1 (Coverley et al., 2005) and now designated CIZ1Δ2p6p8), is compromised in its ability to accumulate at Xi (Figs. 1 C and S1, A–C). Despite efficient nuclear targeting via a conserved nuclear localization signal (NLS) encoded by constitutive exon 7 (functionally validated in human CIZ1; Fig. S1, D–G), this variant does not form assemblies at H3K27me3-marked Xi's with the same efficiency as CIZ1; evident in ∼9% of cycling 3T3 cells after 24 h (Figs. 1 C and S1 C) and 30% of WT PEFs (Fig. S1, A and B). This shows that alternative splicing modulates formation of CIZ1 assemblies at Xi.

**Figure S2.**
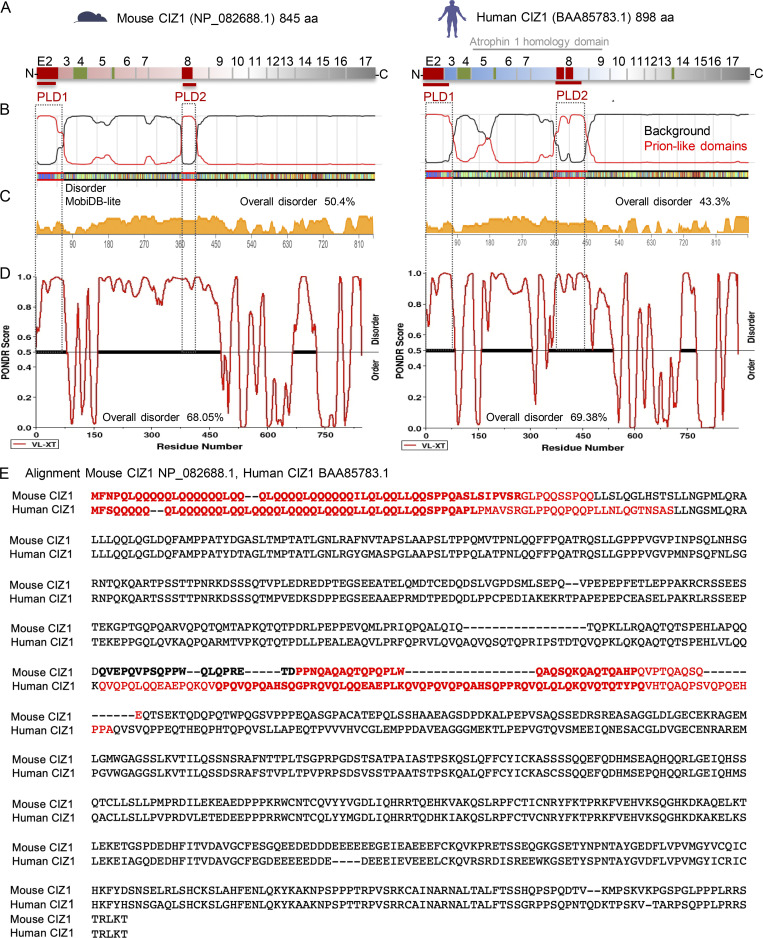
**Similarity between mouse and human CIZ1 alternative splicing, PLDs and structural disorder predictions.** (Related to Fig. 1.) **(A)** Schematics showing mouse and human CIZ1 exon structure, and conditionally excluded exons 2 (Coverley et al., 2005; Warder and Keherly, 2003), 4 (Greaves et al., 2012; Rahman et al., 2007; Warder and Keherly, 2003), 8 (Coverley et al., 2005; Dahmcke et al., 2008), 6 (Coverley et al., 2005; Greaves et al., 2012; Xiao et al., 2012), and 14 (Higgins et al., 2012) in green, or red for those which overlap with PLDs. Exon 1 is untranslated with at least three alternative versions in both human and mouse, and not shown here. Predicted Atrophin 1 homology domain (cl26464) at amino acids 121–482 in human CIZ1 (recently reclassified as cl33720) is shown by gray line. **(B)** PLDs identified by PLAAC (Lancaster et al., 2014) are similar in human and mouse CIZ1. PLD sequences align with alternatively spliced exons 2 (PLD1) and 8 (PLD2) in both mouse and human CIZ1. **(C)** Disorder prediction visualized by MobDB-lite (Piovesan et al., 2021), showing overall 50.4% structural disorder for murine CIZ1 and 43.3% for human CIZ1. **(D)** Disorder prediction by PONDR, showing overall 68.05% structural disorder for murine CIZ1 and 69.38% for human CIZ1. **(E)** Predicted full-length murine (RefSeq accession no. NP_082688.1) and human (BAA85783.1) CIZ1 amino acid sequence, showing glutamine-rich PLD1 and 2 in red and conditionally excluded exons 2 and (part of) 8 in bold.

CIZ1Δ2p6p8 lacks three sequence elements from its N-terminal region (Fig. 1 A), encoded by exon 2 and parts of exons 6 and 8. Those encoded by exons 2 and 8 correspond to PLDs identified by in silico searches for prion-like amino acid composition (PLAAC; Lancaster et al., 2014; Fig. 1 A), and are conserved in human CIZ1 (Fig. S2, A–D). PLD1 encoded by exon 2 comprises short (two- to six-residue) polyQ repeats interspersed with leucine/isoleucine residues (Fig. S2 E), totaling 30 and 29 in human and mouse, respectively. Expansion of CAG repeat elements, which encode glutamine residues, occurs in a group of genes that are linked with neurodegenerative conditions sometimes referred to as polyQ disorders. In unexpanded form, these all normally encode a minimum of 10 consecutive glutamines (Schaefer et al., 2012). Allowing for one mismatch within a run of 10 glutamines, Schaefer et al. (2012) identified a more extensive set of proteins with polyQ tracts that represent 0.3% of the human proteome, enriched in nuclear functions. CIZ1 PLD1 fits this definition, identifying it as a protein with a polyQ tract. PLD2 is also enriched in glutamine residues but is not a polyQ tract. It is however subject to complex alternative splicing that results in at least three length variations in humans (Fig. S2 and Table S1). Both PLDs are subject to conditional exclusion in the naturally occurring CIZ1Δ2p6p8 variant, and are therefore implicated in biologically relevant mechanisms regulating CIZ1 assembly at Xi.

### Requirement for PLD1 in CIZ1 assembly at pre-existing Xi's in WT cells

To directly test which sequences are involved in CIZ1 assembly formation, we created a set of truncation and deletion constructs (Figs. 1 D and S1 C), and screened them by transient transfection into WT female 3T3 cells that contain pre-formed endogenous CIZ1–Xi assemblies. All were expressed and localized to the nucleus, however the N-terminal sequences alone, whether including (N571) or excluding (N442) the three regions spliced out of CIZ1Δ2p6p8, are not sufficient for accumulation at Xi. Similarly, the C-terminal anchor domain (C275) was also dramatically impaired in its ability to accumulate at pre-existing Xi's (Fig. 1 D), evident in a similar proportion of cells as CIZ1Δ2p6p8. For N571 and C275, similar results were reported previously (Ridings-Figueroa et al., 2017). Cotransfection of N- and C-terminal constructs (N571 and C275) did not reconstitute high-efficiency targeting, indicating that both domains are required to be within the same polypeptide.

Mimicking the different alternative splicing events in CIZ1Δ2p6p8 by deleting either partial exon 8 (PLD2) or partial exon 6 individually had no significant effect (Fig. 1 D). However, deletion of exon 2 (PLD1) was sufficient to dramatically suppress assembly in 3T3 cells, and this was confirmed in WT PEFs (passage 3 [p3]; Fig. S1, A and B). Together these data highlight a central role for PLD1 and implicates a polyQ tract in formation of CIZ1 assemblies at Xi, but also shows that it is not sufficient.

### Initiation of new CIZ1 assemblies at Xi in CIZ1-null cells require PLD1 and PLD2

**Figure 2.**
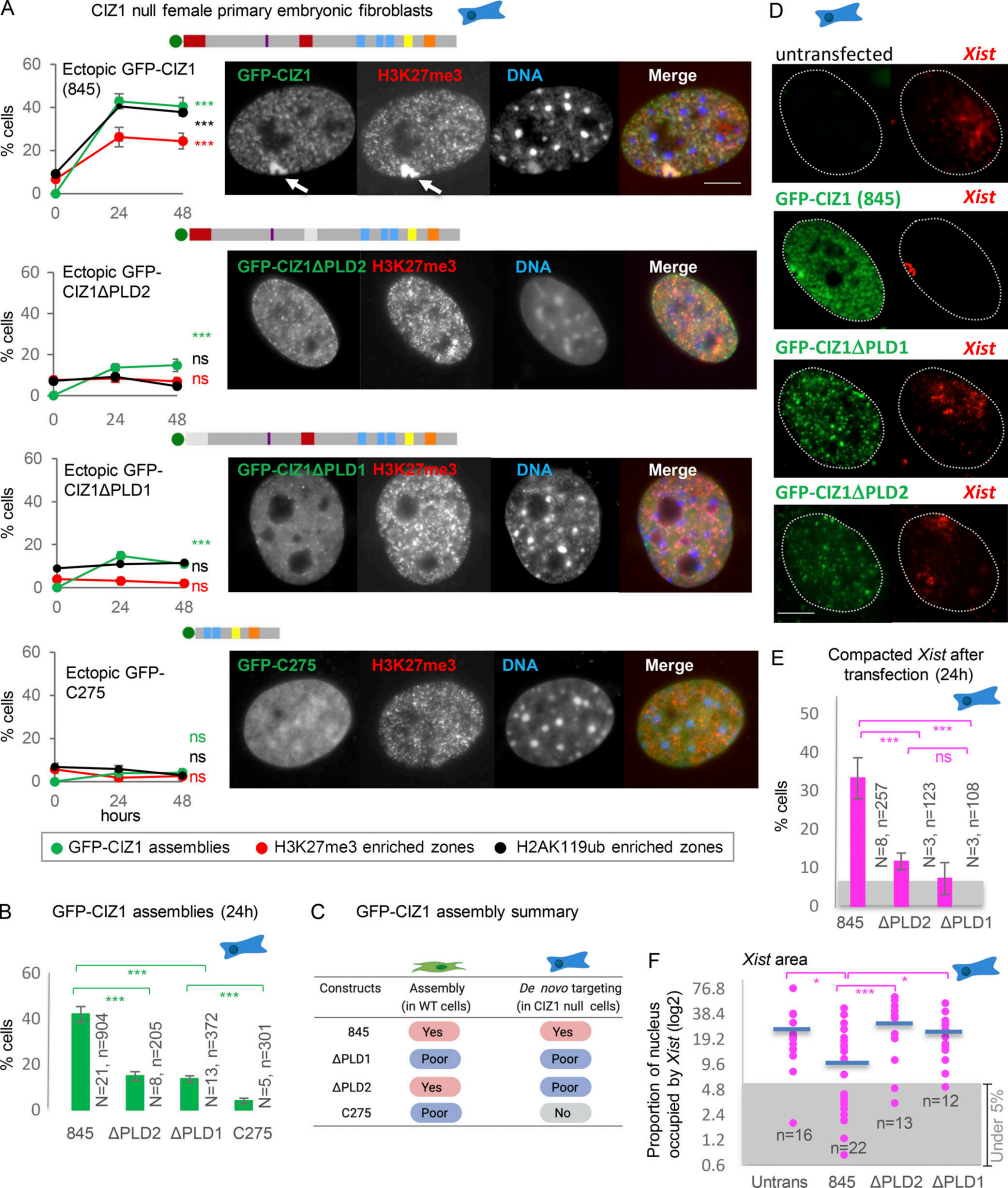
**De novo formation of CIZ1 assemblies and modification of Xi chromatin in CIZ1-null cells. (A)** Frequency of GFP-CIZ1 (green), H3K27me3 (red), or H2AK119ub (black) enriched zones, 24 and 48 h after transfection into CIZ1-null PEFs at p3. Deletion of N-terminal sequences (C275), or absence of either of the polyQ rich PLDs (1–68 or 361–399 in RefSeq accession no. NP_082688.1) excluded by alternative splicing of exon 2 or partial exon 8, reduces the frequency with which GFP-CIZ1 assemblies form and fails to support an increase in the frequency of H3K27me3 and H2AK119ub-enriched chromatin. Results are representative of six experiments with three independent isolates of primary cells (full-length CIZ1), and three experiments with two independent isolates of primary cells (CIZ1ΔPLD1, CIZ1ΔPLD2, and C275 constructs). For CIZ1(845) assemblies *n* at 24/48 h = 749/448; for CIZ1ΔPLD1, *n* at 24/48 h = 283/313; for CIZ1ΔPLD2, *n* at 24/48 h = 205/103; for C275, *n* at 24/48 h = 408/230. Significance indicators (Student’s two tailed *t* test) refer to change over time for H2AK119ub, H3K27me3, and CIZ1 assembly frequency separately. Images show representative nuclei bearing GFP-fusion proteins (green), co-stained for H3K27me3 (red). Bar is 5 μm. **(B)** Summary of all CIZ1 assembly frequency data at 24 h, with significance indicators from five independent CIZ1-null lines, with at least two per construct. *N*, number of replicate transfections; *n*, total number of nuclei inspected. **(C)** Summary of the behavior of the indicated constructs in WT cells and CIZ1-null cells. **(D)** Images showing example nuclei with perimeters defined by DAPI-stained area, after detection of *Xist* by FISH (red). Cells were transfected with the indicated GFP-CIZ1 constructs (green). Bar is 5 μm. **(E)** Frequency of CIZ1-null PEFs with visibly compacted *Xist* signal 24 h after transfection of the indicated forms of GFP-CIZ1. Data are derived from two independent PEF lines (p2) from the indicated number of repeats (*N*) comprising the indicated number of cells (*n*). For CIZ1(845) *N*/*n* = 8/267, for ΔPLD2 *N*/*n* = 3/123, for ΔPLD1 *N*/*n* = 3/108. Significance indicators are derived by Student’s two tailed *t* test. Gray area represents the baseline frequency (before transfection) of compacted *Xist*, derived from the data in F. **(F)** *Xist* area expressed as percentage of nuclear area, from the indicated number of nuclei (*n* = 16/22/13/12 for untransfected cells and CIZ1(845), ΔPLD2, or ΔPLD1 transfected cells, respectively), 24 h after transfection. All transfected cells with nuclear GFP were included (with and without GFP-CIZ1 assemblies), yielding distinct populations for those expressing GFP-CIZ1(845). Gray area represents arbitrary threshold below which *Xist* areas represent <5% of the nucleus and are considered “compact.” Blue bar indicates the mean for each construct, and for untransfected control cells. In all graphs error bars show SEM. *, P < 0.05; ***, P < 0.001.

**Figure S3.**
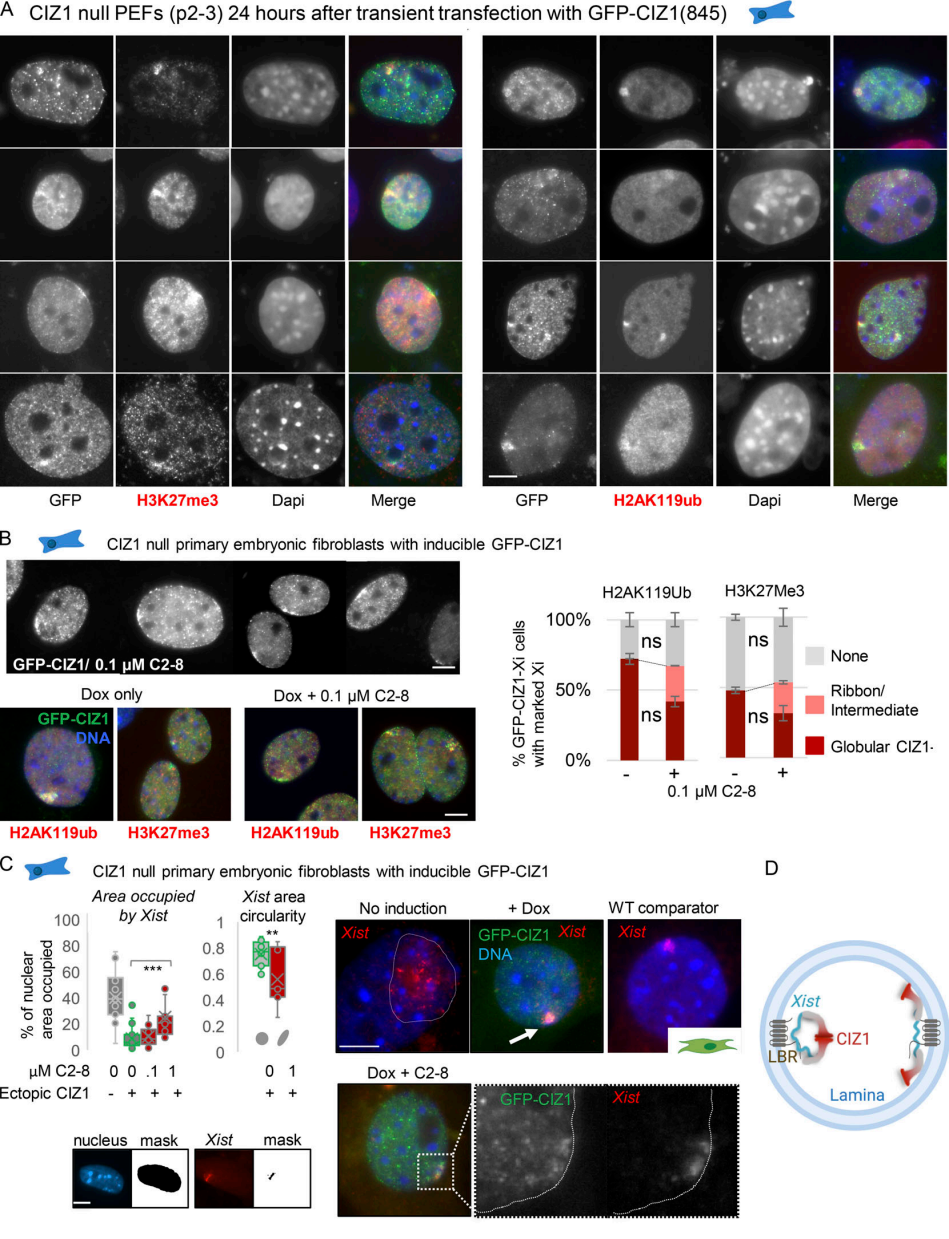
**Ectopic GFP-CIZ1 assemblies, H3K27me3, H2AK119ub, and *Xist*.** (Related to Fig. 3.) **(A)** Assemblage of images showing the range of GFP-CIZ1 assembly shapes and sizes that form 24 h after transient transfection of GFP-845 into CIZ1-null PEFs, all verified by co-staining with H3K27me3 or H2AK119ub. Taken from experiments underpinning the graphs in Fig. 2 A. **(B)** Assemblage of grayscale images showing GFP-CIZ1 in CIZ1-null PEFs, 24 h after doxycycline (dox) induction of integrated vector, all in the presence of 0.1 µM C2-8. Below, co-staining for H3K27me3 or H2AK119ub as indicated. Bar is 5 μm. Right, histograms show the proportion of ectopic GFP-CIZ1 assemblies that co-stain for H2AK119ub or H3K27me3 in female CIZ1-null PEFs (p3), in the presence or absence of C2-8, where ribbons are shown in pink and globular assemblies in red. Gray, no mark. Error bars are SEM from three replicates (where *n* > 100 for each condition and each mark); ns (not significant) indicates that modification of Xi chromatin persists in assemblies whose structure is affected by C2-8. Comparisons are by Student’s two tailed *t* test. **(C)** Left, graph shows the effect of C2-8 on the nuclear area occupied by *Xist* upon induction of GFP-CIZ1 in CIZ1-null PEFs (24 h after dox induction of integrated vector). Results are expressed as percentage of nuclear area, calculated from area masks generated in Fiji. Uninduced, *n* = 19; induced, *n* = 33; with 0.1 µM C2-8, *n* = 21; with 1 µM C2-8, *n* = 19. Right, shape of *Xist* areas in GFP-CIZ1–expressing cells with and without 1 µM C2-8, expressed as degree of circularity of *Xist* masks returned by Fiji. Example images show retention of *Xist* (red) upon induction of GFP-CIZ1 and the formation of de novo CIZ1 assemblies (green), in the absence and presence of C2-8, with high magnification; separate views of CIZ1 and *Xist* in a linearized assembly in grayscale. A WT cell showing normal *Xist* cloud is shown for comparison. Bars are 5 μm. **(D)** Illustration of possible antagonistic effect of polyQ aggregation on the interaction between Xi chromatin and the nuclear lamina. **, P < 0.01; ***, P < 0.001.

In primary fibroblasts lacking CIZ1, *Xist* is not captured at Xi but is dispersed throughout much of the nucleus (Ridings-Figueroa et al., 2017), and both H3K27me3 and H2AK119ub are typically absent from Xi chromatin (Stewart et al., 2019). It should be noted that, during prolonged culture, both marks re-emerge in CIZ1-null culture-adapted fibroblasts, co-incident with upregulation of EZH2, so the present analysis is carried out exclusively in early passage populations (p2–p4). These typically have a low frequency of already mark-enriched Xi chromatin; ∼5% of cells depending on strain and “age.” It is against this baseline frequency that change is measured during a 24–48 h window of expression of CIZ1, or derived deletion constructs. Re-expression of full-length CIZ1 (845) from an inducible vector (Ridings-Figueroa et al., 2017), or by transient transfection (Fig. 2, A and B) supports de novo assembly of typically one large CIZ1 assembly per cell within 24 h, accompanied by enrichment of H3K27me3 and H2AK119ub-marked chromatin (Figs. 2 A and S3 A). Thus, ectopic CIZ1 is capable of establishing features of Xi chromatin in fibroblasts de novo. However under the same conditions, GFP-CIZ1ΔPLD1(Δexon2) is impaired in its ability to initiate new assemblies (present in 15% of cells that express it, compared to 43% for full-length CIZ1 at 24 h), and completely fails to support new modification of Xi chromatin, evidenced by no increase in the frequency of H3K27me3 or H2AK119ub-enriched chromatin over time. This shows that CIZ1 PLD1 is required for repressive modification of Xi chromatin in differentiated fibroblasts.

However, more surprisingly the PLD2 deletion (partial exclusion of exon 8) was similarly impaired in assembly formation, and in facilitating enrichment for H3K27me3 or H2AK119ub (Fig. 2 A). This contrasts with its behavior in WT cells (Figs. 2 C and 1 D), indicating that PLD2 is required specifically for de novo formation of new assemblies. We also evaluated GFP-C275 in CIZ1-null cells and saw little evidence of assembly formation or any enrichment of H3K27me3 and H2AK119ub (Fig. 2 A), indicating that its residual capacity to assemble at Xi observed in WT cells requires the prior assembly of endogenous CIZ1. Therefore, the MATR3 domain (smart00451) and Jazz-type Zinc-finger (pfam12171) with predicted RNA-binding capacity are not sufficient to support CIZ1 assembly.

Finally, we compared the effect on *Xist*, which is not normally retained as a compact assembly surrounding Xi in primary fibroblasts lacking CIZ1, but is dispersed throughout much of the nucleus (Ridings-Figueroa et al., 2017; Sunwoo et al., 2017). In CIZ1-null cells expressing ectopic GFP-CIZ1(845), CIZ1 assembly formation was indeed accompanied by compaction of *Xist* (Fig. 2 D), both in terms of the number of cells that respond (Fig. 2 E) and the average area of the nucleus occupied by *Xist* (Fig. 2 F). For both measures, all transfected (GFP-expressing) cells were included in the analysis, 42% of which typically have formed CIZ1 assemblies by 24 h. These results are consistent with existing data, which suggests co-recruitment (of CIZ1 and *Xist*; Ridings-Figueroa et al., 2017; Sunwoo et al., 2017). In contrast to GFP-CIZ1(845), for both GFP-CIZ1ΔPLD1 and GFP-CIZ1ΔPLD2, we saw no significant effect on *Xist* retention; the background frequency of nuclei with compact signal did not increase upon expression of these deletion constructs, and the area occupied was similar to untransfected cells (Fig. 2 F). Thus, GFP-CIZ1 assembly capability correlates with retention and compaction of *Xist*, and suggests that *Xist* capture is dependent on PLD1 and PLD2 in differentiated fibroblasts.

### PolyQ-mediated interaction

**Figure 3.**
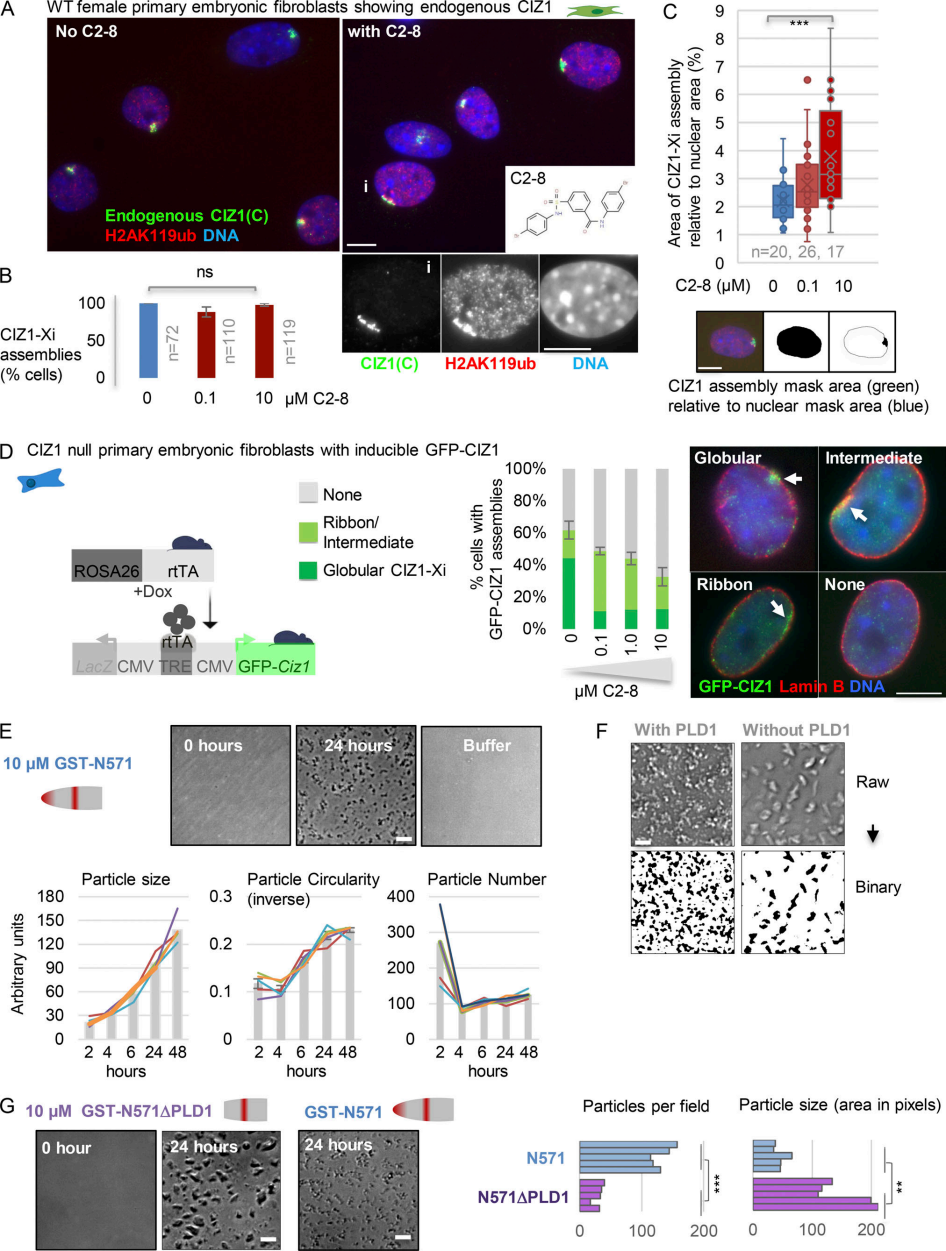
**PolyQ-mediated assembly. (A)** Example images showing endogenous CIZ1 (C-term, green) and H2AK119ub (red) in female WT PEFs at p3, without (left) and with (right) incubation with polyQ aggregation inhibitor C2-8 (inset) for 24 h. DNA is blue. Below, example nucleus (i) with lamina-associated ribbon-like CIZ1 assembly. Bar is 5 μm. **(B)** Histogram showing frequency of CIZ1 assemblies derived from two cycling population of WT PEFs (p3) without (blue) and with (red) exposure to C2-8, where the number of nuclei inspected (*n*) is 72, 110, and 119 for control and 0.1 and 10 µM C2-8, respectively. **(C)** Box-and-whisker plot showing area of CIZ1 assemblies (green signal in images), calculated as a proportion of nuclear area without (blue) and with (red) C2-8, generated using image masks in Fiji (below; bar is 5 μm). Data are representative of two experiments with independent WT primary cell isolates, where *n* = 20, 26, and 17 for control and 0.1 and 10 µM C2-8, respectively. **(D)** Left: Schematic of transgenes used to create doxycycline (dox)-inducible expression of full-length GFP-CIZ1(845) in PEFs derived from CIZ1-null mice (Ridings-Figueroa et al., 2017). Tet-responsive element (TRE), CMV promoter (CMV), and reverse tetracycline transcriptional activator (rtTA). Histogram shows frequency of globular or ribbon-like GFP-CIZ1 assemblies (shades of green) or absence of assemblies (gray) when cycling CIZ1-null PEFs at p3 were exposed to the indicated concentrations of C2-8 throughout a 48-h induction period. Right: Example images showing accumulation of GFP-CIZ1 (green) at Xi, as either a typical globular structure or an elongated ribbon-like structure associated with the nuclear lamina. Nuclei are counterstained for lamin B (red); DNA is blue. Bar is 5 μm. *N* = 3, with *n* > 150, per condition. **(E)** Phase-contrast images showing CIZ1 N-terminal fragment N571 (10 µM) at the start and end of a 24-h incubation. Bar is 10 μm. Below, quantification of particle size, number, and circularity (expressed as inverse on a scale of 0–1) over time for samples of N571 at 10 µM. Histograms show mean of five samplings where each contains in excess of 50 particles, and individual values as line graphs. **(F)** Illustration showing conversion of phase-contrast images to binary format to derive quantitative information on assemblies using Fiji (Schindelin et al., 2012). Bar is 10 μm. **(G)** Comparison of assemblies formed by N571 and N571ΔPLD1 at 10 µM after 24 h. Bar is 10 μm. Right, quantitation of binary images showing average particle number and particle size per field. Significance by Student’s two tailed *t* test; **, P < 0.01; ***, P < 0.001. In all graphs, error bars show SEM.

The requirement for PLD1 for accumulation at Xi in WT cells led us to question whether interfering with polyQ-mediated interactions would impact characteristics of Xi implicated in maintenance of gene expression. Aberrant polyQ-mediated protein aggregation is well documented in relation to neurodegenerative disorders such as Huntington’s disease, in which small molecule inhibitors have been trialed therapeutically. One such molecule, a cell-permeable amidosulfonamide compound, C2-8, inhibits polyQ aggregation in vivo when used in the micromolar range (Zhang et al., 2005). C2-8 altered the degree of compaction (size of CIZ1 assemblies expressed as proportion of the nucleus) but not the frequency of endogenous CIZ1 assemblies (proportion of cells with an assembly) in WT PEFs (Fig. 3, A–C) and had an even more striking dose-dependent effect when applied to CIZ1-null PEFs during doxycycline-induced GFP-CIZ1 transgene expression (Fig. 3 D). Typically ectopic GFP-CIZ1 forms globular assemblies within 24 h of induction (Ridings-Figueroa et al., 2017), but their compaction, shape and frequency were affected by C2-8, whereby in some cells no GFP-CIZ1 assemblies were observed, or if present were more likely to appear as irregular shapes including extended ribbons adjacent to the inner face of the nuclear lamina (Fig. 3 D). In both endogenous (Fig. 3 A) and ectopic CIZ1 contexts (Fig. S3 B), we saw no evidence of reduced accumulation of H3K27me3 or H2AK119ub, which was evident in cells even with flattened ribbon-like CIZ1 assemblies. Moreover, like CIZ1 assemblies, *Xist* territories were less compact and more irregular in shape after expression of CIZ1 in the presence of C2-8 (Fig. S3 C). The effects of C2-8 in these experiments could be indirect, via interference with polyQ domains of other proteins, nevertheless, it is clear that polyQ-mediated interactions influence the shape and structure of the CIZ1-*Xist* assembly. We speculate that polyQ-mediated interactions could antagonize the reported interaction between *Xist* and lamin B receptor (Chen et al., 2016) that normally anchors Xi at the nuclear periphery (Fig. S3 D) and therefore might influence the *Xist* and CIZ1-dependent transient internalization of Xi reported previously (Stewart et al., 2019; Zhang et al., 2007).

### CIZ1 forms condensates in vivo and self-assemblies in vitro

Much of CIZ1 (not just its PLDs) is predicted to be structurally disordered (Fig. S2, C and D), a defining feature of proteins which undergo phase separation in vivo (Alberti, 2017; Shin and Brangwynne, 2017). Even in male murine fibroblasts, uncomplicated by events at Xi, CIZ1 coalesces into large subnuclear assembles, and this inherent propensity to self-associate is modulated by alternative splicing (Ainscough et al., 2007; Rahman et al., 2007). Similarly, in human cells, ectopic full-length GFP-CIZ1 initially forms nuclear foci similar to endogenous CIZ1 but then coalesces inside the nucleus over time, and ultimately kills host cells (Higgins et al., 2012). Here, we extend these observations and show that exclusion from the high-RNA environment of the nucleus, via mutation of its NLS, influences condensate formation so that full-length CIZ1 forms large aggregates in the cytoplasm immediately upon expression (Fig. S1 G). To explore and better quantify these behaviors observed inside cells, we purified GST-N571 and GST-N571 lacking PLD1 or PLD2 and compared their properties in vitro.

**Figure S4.**
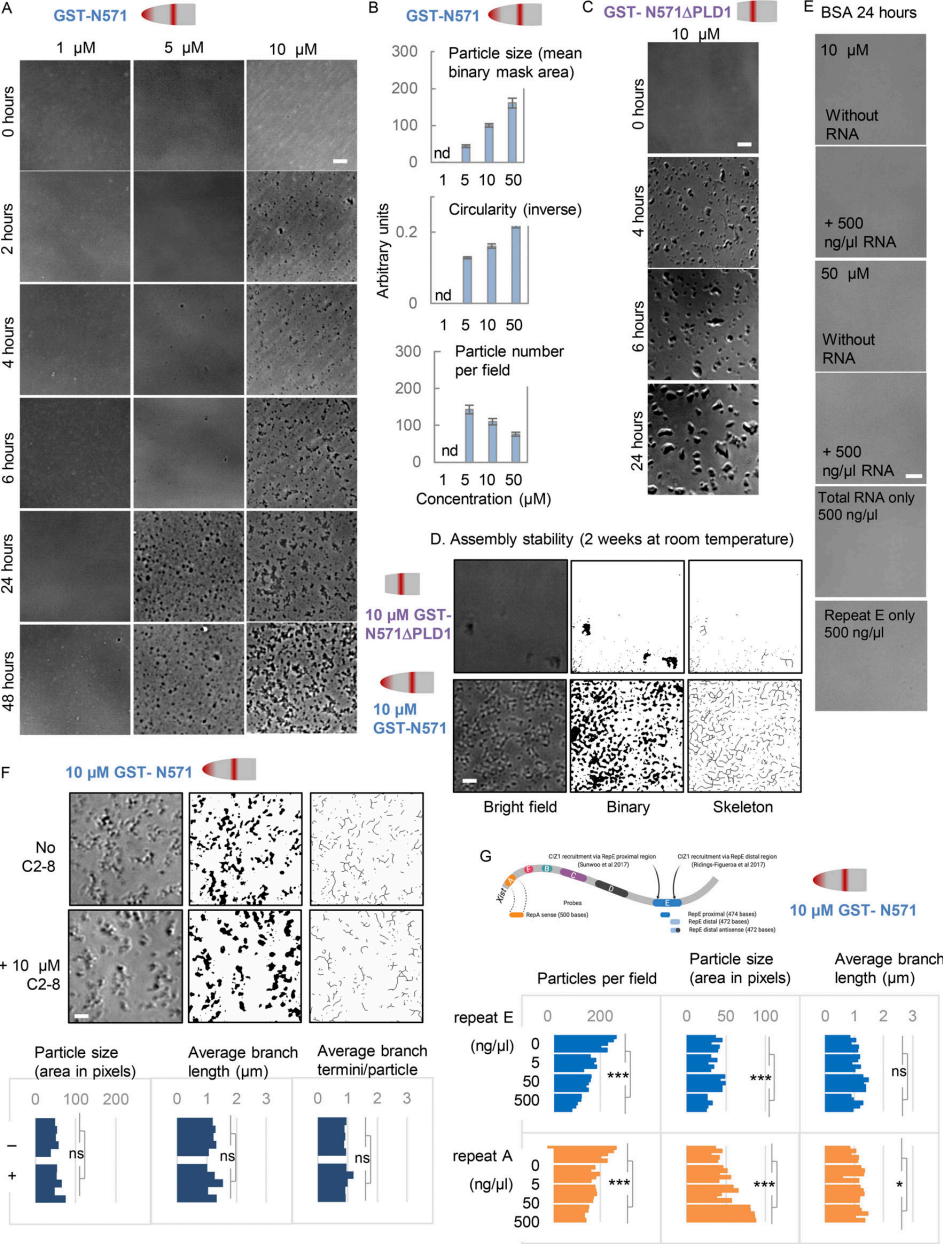
**Supporting in vitro assembly data.** (Related to Figs. 3 and 4.) **(A)** Example phase contrast images of N571 (left) at the indicated times and concentrations, showing emergence of mesh-like assemblies. **(B)** Histograms show the mean size, circularity, and number of particles formed in samples of N571, incubated at the indicated concentrations for 24 h. Error bars show SEM from five samplings, where each contains >50 particles. nd, not determined. **(C)** Example phase-contrast images of N571ΔPLD1 incubated at 10 µM for the indicated times. **(D)** N571 and N571ΔPLD1 at the end of a 2-wk incubation at room temperature (sealed and dark), visualized after conversion to binary format and skeletonization. **(E)** Control protein (BSA) alone and with RNA (total cellular RNA from female cultured fibroblasts), in vitro transcribed repeat A and E proximal RNA alone, all imaged after 24 h at the indicated concentrations. **(F)** N571 after 24 h without (upper) and with (lower) inclusion of 10 µM C2-8. Bar is 10 μm in all cases. Below, particle and skeleton parameters (Materials and methods), from five samplings where each contains >50 particles, are not affected by C2-8 under these conditions. **(G)** As in Fig. 4 A except that *Xist*-derived RNA fragments repeat E proximal (orange) and repeat A (blue) are tested for their effect on N571, at the indicated concentrations. Graphs show assembly parameters derived from bright-field images taken at 24 h, with significance by Student’s two tailed *t* test. *, P < 0.05; ***, P < 0.001.

Typically, phase separation gives rise to spherical condensates, whereas CIZ1 N571 assemblies resemble an irregular fibrillar network, which forms in a manner dependent on time and concentration (Fig. 3 E and Fig. S4, A and B). Standardized quantification of phase-contrast images after conversion to binary format (Fig. 3 F) showed that N571 assemblies (at 10 µM) are first detectable at 2 h and continue to grow up to 48 h (Fig. 3 E). Particle number decreased as size increased, and this was accompanied by a decrease in circularity. Thus in vitro, N571 undergoes spontaneous self-interaction (independent of RNA) to form microscopically visible assemblies of mean overall length 6.2 μm (± 0.6) at 24 h.

In contrast, the structures formed by N571ΔPLD1 were less complex and did not resemble a network. Quantification of binary images (10 μM at 24 h) showed significantly fewer particles per field that were larger in size than N571 (Figs. 3 G and S4 C). Moreover, while PLD1-containing N571 fibrils remained intact, even after 2 wk at room temperature, N571ΔPLD1 assemblies largely dissipated during this time (Fig. S4 D). Although we were not able to record an effect of C2-8 on assembly formation in vitro (Fig. S4 F), this data nevertheless implicate PLD1 in assembly structure and stability, because its absence causes a shift from highly stable network-like assemblies to discrete but transient entities.

### Effect of RNA

**Figure 4.**
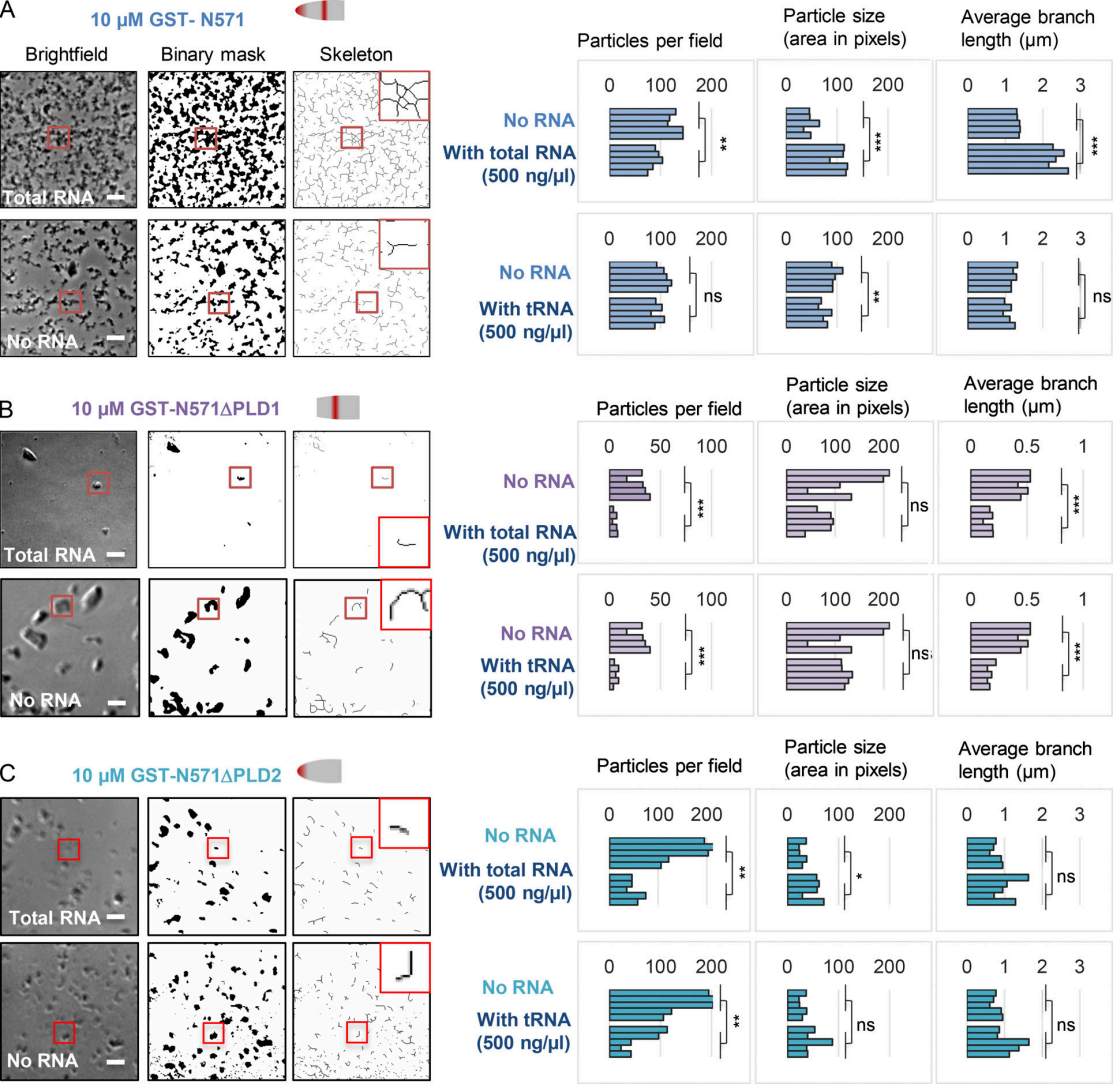
**PLD1-dependent self-assembly and modulation by RNA. (A)** Example bright-field images showing N571 mesh-like assemblies, image conversion to binary format, and image skeletonization using Fiji. Inset to skeleton: High-magnification view of area selected in red square, illustrating branch structure. Bar is 10 μm. Left: Quantitation of particle or skeleton-derived parameters as indicated, in five individual samplings each containing in excess of 50 particles, showing the effect of inclusion of 500 ng/μl total cellular RNA from female murine 3T3 cells, or 500 ng/μl tRNA, compared by Student’s two tailed *t* test. Total RNA increases particle size and branch length. **(B)** As in A, but for N571ΔPLD1. **(C)** As in A, but for N571ΔPLD2. Both types of RNA reduce particle formation with these CIZ1 variants. *, P < 0.05; **, P < 0.01; ***, P < 0.001.

Some PLD proteins, such as Whi3, are capable of condensing on their own (Zhang et al., 2015), but multivalent interactions between PLD proteins and RNA typically modulate the properties of condensates, by both driving their formation and controlling morphology (Langdon et al., 2018; Maharana et al., 2018), while at high concentrations such as those in the nucleus (Maharana et al., 2018) RNA can buffer against coalescence (Jain and Vale, 2017; Maharana et al., 2018; Yamazaki et al., 2018). We tested the effect of total cellular RNA and also tRNA (at 500 ng/μl) on N571 assemblies formed in vitro (10 μM at 24 h), and recorded distinct effects that were quantified after skeletonization of binary images (Fig. 4 A). For N571, neither RNA suppressed assembly formation, but total cellular RNA led to a quantifiable increase in particle size (Fig. 4 A), manifested as an increase in average total pixels per particle and the average maximum length of branches per skeleton. In contrast, tRNA did not drive a change in these parameters. Thus, total cellular RNA appears to promote polymerization of CIZ1, and possibly the formation of bridges between assemblies, but does not significantly increase their complexity. Neither RNA alone, nor BSA (in the presence or absence of RNA) formed microscopically visible assemblies under the same conditions (Fig. S4 E).

Similar analysis of N571ΔPLD1 and N571ΔPLD2 showed that they neither grow their size or branch structure in the presence of total cellular RNA and, in general, inclusion of RNA has a suppressive effect on assemblies formed from both proteins (Fig. 4, B and C). This suggests that both PLDs are likely to contribute to assembly size and structure, inside the RNA rich nucleus of cells.

### CIZ1 binds *Xist*

**Figure 5.**
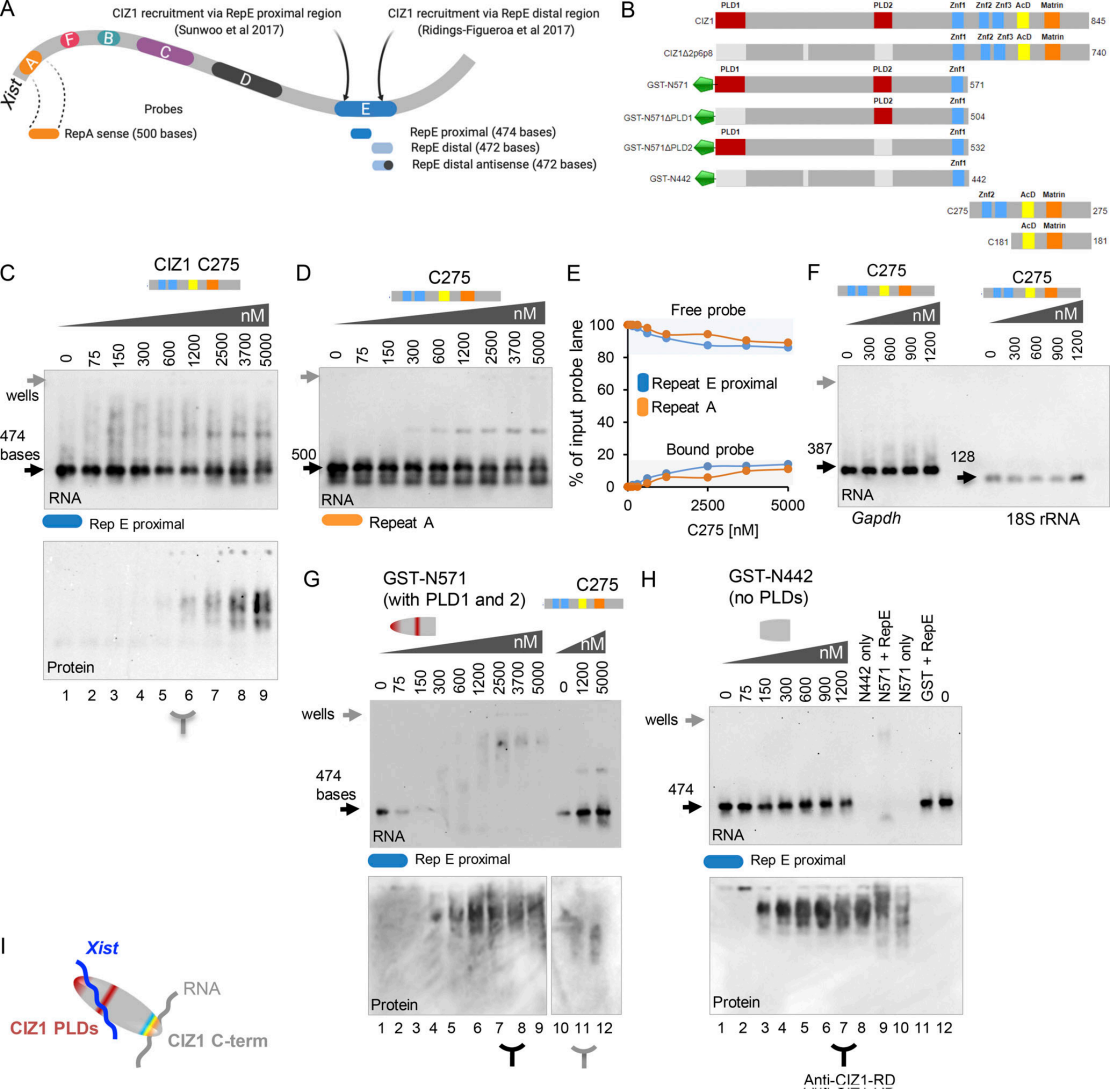
**Multivalent interaction between CIZ1 and RNA. (A)** Schematic representation of repeat elements in mouse *Xist* showing repeats A, F, B, C, D, and E (Nesterova et al., 2001) and regions of repeat E thought to be involved in CIZ1 recruitment to Xi. Proximal and distal, sense and antisense, in vitro–transcribed RNA probes made from *Xist* repeat E, are indicated below, as well as a comparison probe made from repeat A. **(B)** Schematic representation of domain architecture of mouse CIZ1 and the truncated versions used here. PLD1 and PLD2 in mouse CIZ1 (NP_082688.1) are red, ZnF1-3 (zinc finger domains 1, 2 and 3, also annotated as Jasmonate ZIM-domain by Conserved Domain Database) are blue; acidic domain (AcD) is yellow; and Matrin 3 domain is orange. **(C and D)** Representative EMSAs showing binding of recombinant CIZ1 C-terminal fragment C275 with *Xist* repeat E proximal probe and *Xist* repeat A. Below, CIZ1 Western blot of the EMSA with repeat E. **(E)** Quantitation of interaction between C275 and repeats E and A, showing proportion of input probe lost from the free probe position or gained in the shifted position (bound), expressed as percentage with SEM calculated from three independent EMSA experiments, each with 0.3 nM RNA probe. C275 has similar low affinity for both probes. **(F)** No interaction between C275 and non-*Xist* control probes (*Gapdh* or 18S rRNA) up to 1,200 nM. **(G and H)** EMSAs showing binding profile of CIZ1 N571 (plus C275 shown for comparison) and N571Δ2p6p8 (also known as fragment N442, which lacks exons 2p6p8), with N571 and free GST shown for comparison, tested using repeat E proximal probe. The image in G is reproduced in Fig. S5 C with demarcation of areas used for quantification. Below, Western blots of the EMSAs, including N571 (1,200 nM) without (lane 10) and with (lane 9), repeat E proximal probe. **(I)** Schematic showing CIZ1 interacting with *Xist* via its PLD-containing N-terminal RNA interaction domain, and unspecified RNA via its C-terminal interaction domain.

**Figure S5.**
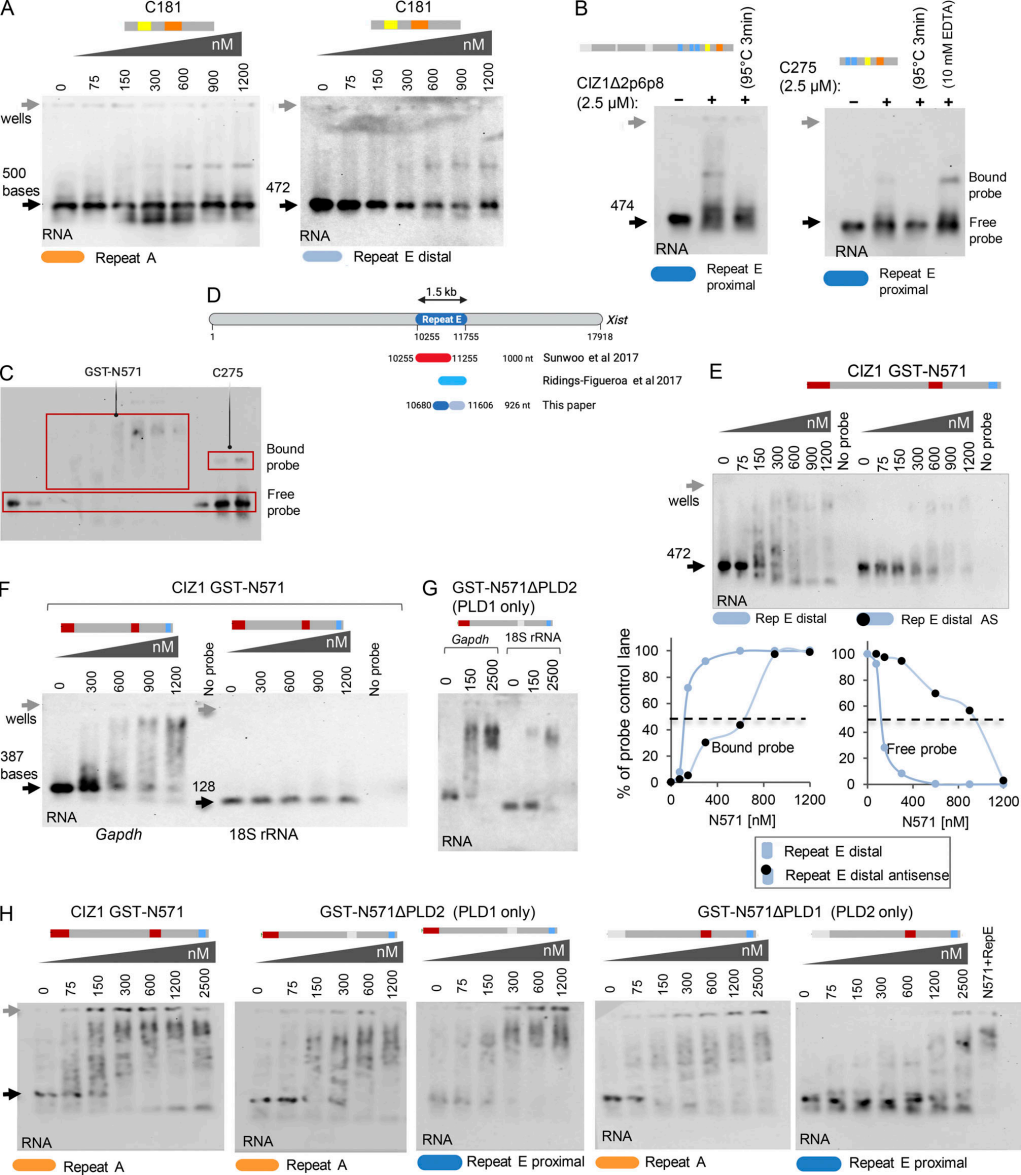
**Supporting EMSA data.** (Related to Figs. 5 and 6.) **(A)** C-terminal fragment C181 interaction with *Xist* repeat A and repeat E (distal sense) probes respectively (analysis as for C275 in Fig. 5, C–F). **(B)** EMSA showing CIZ1Δ2p6p8 and C275 protein fragments binding to proximal repeat E RNA. Binding is lost upon heating to 95°C for 3 min before sample loading, but addition of 10 mM EDTA enhances binding. A 6.5-nM RNA probe was used in all lanes. **(C)** Example EMSA (reproduced from Fig. 5 G) showing which RNA fractions (red rectangles) were used to generate graphs in main figures. **(D)** Map showing mouse *Xist* (RefSeq accession no. NR_001463.3) and 1.5 kb long *Xist* repeat E region (highlighted in blue). Repeat E regions implicated in CIZ1 recruitment as used by Sunwoo et al. (2017) (red) and Ridings-Figueroa et al. (2017) (light blue) are indicated. Repeat E probes proximal (dark blue) and distal (gray) used in this paper to test for direct CIZ1 binding are indicated below. **(E)** Comparison of *Xist* repeat E distal sense and antisense RNA probes and their interaction with N571. Below is graph comparing fraction of bound and free RNA for sense and antisense E probes, indicating clear sequence preference. **(F)** EMSA showing N571 binding with *Gapdh*, but not 18S rRNA. **(G)** EMSA showing binding of N571 lacking PLD2 (N571ΔPLD2) with both *Gapdh* and 18S rRNA. **(H)** Further example EMSA experiments, as indicated, to support Fig. 6. Unless otherwise stated, RNA concentration was 0.3 nM and all experiments were performed three times, carried out on different days.

*Xist* lncRNA is defined by a series of repeat motifs (A–F in the mouse, Fig. 5 A) that interact with RBPs with functions in gene silencing (Monfort and Wutz, 2020; Nesterova et al., 2001). Our data and that of others indicate a functional relationship between CIZ1 and *Xist*, specifically its repeat E element (Ridings-Figueroa et al., 2017; Sunwoo et al., 2017), which consists of C/U/G-rich tandem repeats of 20–25 nucleotides long, over approximately 1.5 kb at the beginning of exon 7. However, although high-resolution imaging shows that CIZ1 particles and *Xist* are in close proximity (Ridings-Figueroa et al., 2017; Rodermund et al., 2021), and CIZ1 was identified as a candidate *Xist*-interactor in vivo by comprehensive identification of RNA-binding proteins by mass spectrometry (Chu et al., 2015), none of the existing data demonstrate their direct binding. Moreover, the two independent deletion studies of *Xist* (Ridings-Figueroa et al., 2017; Sunwoo et al., 2017), implicate different portions of repeat E in recruitment of CIZ1 to Xi. Therefore to test for a direct interaction, we generated in vitro–transcribed *Xist* RNA probes from different regions of repeat E, with repeat A for comparison (Fig. 5 A and Table S2) plus a set of controls, for use in electrophoretic mobility shift assays (EMSAs) with recombinant CIZ1 proteins (Fig. 5 B). We detected multiple discrete modes of binding by studying the N-terminal and C-terminal CIZ1 fragments separately. Using either *Xist* repeat A, which in vivo is not implicated in CIZ1 recruitment to Xi (Ridings-Figueroa et al., 2017), or the proximal (sense) region of repeat E which is implicated, we detected formation of a stable discrete complex with recombinant C275 fragment. Affinity is similarly low for both probes, so that only 10–15% of input probe was complexed even under protein concentrations as high as 5 µM (Fig. 5, C–E). Binding is first evident at 600 nM, and in this range C275 did not interact with *Gapdh*, or with 18S rRNA (Fig. 5 F), indicating that its interaction with RNA is not promiscuous. To ask whether interaction with *Xist* is mediated by the zinc finger domains in C275, we created a shorter construct encompassing just the C-terminal 181 amino acids of CIZ1 (Fig. 5 B), which lacks all three C2H2 zinc fingers. Results were similar to those with C275, indicating that the Zinc fingers are not required for this C-terminal interaction with *Xist* (Fig. S5 A). CIZ1Δ2p6p8, which contains the whole C275 sequence, also formed a stable complex with *Xist* repeat E to a similar extent as C275 (Fig. S5 B).

Under the same conditions, GST-N571 (containing both PLDs) formed stable complexes with repeat E (Fig. 5 G), and with much higher affinity than C275, so that essentially all the input probe was complexed and shifted in the low µM range. Notably, N571 did not form a discrete RNA-protein complex but instead produced a broad smear (Fig. S5 C) indicative of a complex array of nucleoprotein species (Grigoryev and McGowan, 2011). This could reflect protein–protein interaction, or multiple and variable numbers of CIZ1 molecules interacting with each RNA molecule, or a combination of both. Visualization of input protein by Western blot (Fig. 5 H, lower) shows that N571 exists as more than one species under native conditions (lane 10), and this is modulated by exposure to repeat E (lane 9). No interaction was detected when GST was tested alone (Fig. 5 H). In contrast to GST-N571, GST-N442 (N-terminal fragment lacking both PLD1 and PLD2) completely failed to shift repeat E (Fig. 5 H) highlighting the potential for conditional inclusion of CIZ1’s PLD domains to modulate its interaction with *Xist*. This analysis of N- and C-terminal domains in isolation from each other therefore illustrates at least two independent RNA interaction interfaces (Fig. 5 I), both competent to interact with *Xist*.

### A complex relationship with repeat E

**Figure 6.**
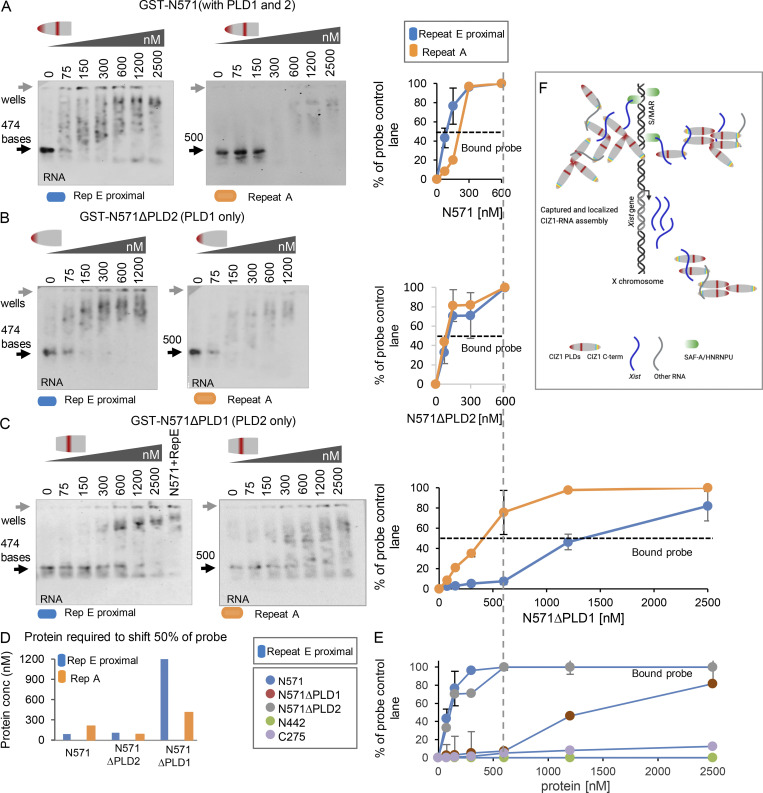
**PLD1 and PLD2 confer preference for repeat E. (A)** Example EMSA showing a concentration gradient of CIZ1 N571 with *Xist* repeat E proximal probe on left; repeat of A on right, and graph showing fraction of probe bound and shifted at the indicated protein concentrations. Means were derived from three independent experiments carried out with 0.3 nM RNA probe. Dotted line shows where a shift of 50% of input probe is achieved. Example of area used for quantitation is illustrated in Fig. S5 C. **(B)** As in A, except with N571ΔPLD2 (contains PLD1 but not PLD2). **(C)** As in A, except with N571ΔPLD1 (contains PLD2 but not PLD1). **(D)** Histogram illustrating the effect of PLD deletion on the concentration (conc) of N571, or derived variant, required to shift 50% of input repeat E or repeat A probes. **(E)** Comparison of the effect of CIZ1 protein fragments shown in Fig. 5 B, on mobility of repeat E proximal probe, expressed as fraction of probe shifted across the protein concentration gradient. **(F)** Model showing multivalent interaction between CIZ1 and RNAs including *Xist,* in the vicinity of Xi. SAF-A–anchored *Xist* at local S/MARS captures CIZ1 via high-affinity PLD-mediated interaction to initiate formation of a protein–RNA matrix, which is amplified by interaction with other RNAs, and PLD1-dependent self-interaction between CIZ1 molecules. In all graphs, error bars show SEM.

We next compared the interaction between GST-N571 and *Xist* repeats E and A. Unlike C275, in which we detected no difference in affinity (Fig. 5 E), GST-N571 showed a moderate but reproducible preference for repeat E (and overall higher affinity for both probes; Fig. 6 A). A half-maximal shift of repeat E was achieved at half the concentration of protein (100 nM) than was required for repeat A (200 nM). Similar evidence of specificity was recorded when comparing the distal portion of repeat E to its anti-sense sequence (Fig. S5, D and E). N571 also formed a complex with *Gapdh* to a similar extent as anti-sense distal E, but did not interact with 18S *rRNA* (Fig. S5 F). These data show that the N-terminal CIZ1 RNA interaction domain has a preference for the repeat E sequence, that it can interact with either proximal or distal elements within repeat E, but that it can also interact with other RNAs. Taken together, these two-component EMSA studies also indicate direct multivalent interaction between CIZ1 and RNA, and show a weak but measurable preference for repeat E.

Notably, in in vitro assembly assays *Xist* repeat E and repeat A impacted differently on the formation of PLD1-dependent N571 networks (Fig. S4 G). At the same RNA concentration (500 ng/ul) and time point (24 h), repeat A drove an increase in assembly size similar to total RNA, while repeat E did not. In fact, repeat E caused a significant measurable reduction in assembly size, further indicating a sequence specific relationship with CIZ1.

Together these two data sets indicate a complex relationship with repeat E. While it is an essential determinant of CIZ1 assembly formation at Xi in vivo (Ridings-Figueroa et al., 2017; Sunwoo et al., 2017), interaction cannot be assumed to be entirely positive, and may in fact play a modulatory role.

### Contribution of PLD1 and PLD2

GST-N571Δ2p6p8 has neither PLD1 nor PLD2 and failed to interact with any RNA probe tested (Fig. 5 H), so we next tested the contribution of the PLD domains in separate deletion fragments. Deletion of PLD2 resulted in loss of specificity for repeat E so that interaction with repeat E and A probes was similarly high, with half maximal shift achieved in the 100 nM range (Fig. 6 B). Thus, PLD2 appears to contribute a degree of selectivity by reducing affinity for repeat A. Consistent with a restraining role, alone among all the CIZ1 protein fragments we tested, N571ΔPLD2 also gained the ability to interact with 18S RNA (Fig. S5 G). In contrast, N571ΔPLD1 was massively impaired in its ability to interact with repeat E or repeat A, although it did retain reproducibly more affinity for repeat A than E (Fig. 6 C). Direct comparison of the protein concentrations required to shift 50% of input probe illustrates the contribution of PLD1 to RNA interaction affinity (Fig. 6 D), and comparison of all five different protein species interaction with repeat E underlines the contribution of both PLD1 and PLD2 to binding (Fig. 6 E). Together these data implicate PLD1 in high-affinity interaction, most dramatically for repeat E, and PLD2 in dampening interaction, except with repeat E. We therefore suggest that PLD2 performs a gatekeeper function imparting some specificity on the promiscuity of PLD1 (Fig. 6 F).

## Discussion

The evidence presented here allows us to draw several conclusions about the behavior of CIZ1 and to relate that to its function at Xi in differentiated murine fibroblasts. First, PLD2 is dispensable for formation of large subnuclear assemblies at Xi's that are already populated by endogenous CIZ1, but is essential to support initiation of new assemblies in cells devoid of endogenous CIZ1. PLD2 appears to impact specificity for repeat E by dampening CIZ1’s interaction with other RNA sequences, mediated by the otherwise promiscuous binding domain PLD1. Thus, inside cells the resulting specific and direct interaction with *Xist* repeat E could seed the formation of CIZ1 assemblies at *Xist*-rich locations. Inside cells specific RNAs are known to seed protein assemblies controlling where condensation takes place (Zhang et al., 2015), the most well-known example being the nuclear paraspeckles that form around the lncRNA *Neat1* (Hennig et al., 2015; Protter et al., 2018), and indeed *Xist*, which is now known to seed a multiprotein condensate near its transcription site (Pandya-Jones et al., 2020).

A functional relationship between CIZ1 and *Xist* repeat E was previously evidenced by two studies that used deletions to implicate either its proximal (Sunwoo et al., 2017) or distal (Ridings-Figueroa et al., 2017) part. Here we independently tested two regions of repeat E and detected similar interaction with both in vitro, suggesting that any part may be sufficient to support interaction with CIZ1, and that not all of the constituent repeats are required. Despite a measurable preference for repeat E, the data also show interaction with repeat A, anti-sense E, and unrelated *Gapdh* RNA probes of similar length. Thus, the distinction is not strong in vitro, implying that relatively small differences in affinity for repeat E over other RNAs may be sufficient to capture CIZ1, or that additional factors may contribute to specificity in vivo. Weak specificity is in line with other analyses that shows that even the prion like RBP FUS, which nucleates around *Neat1*, exhibits unspecific RNA binding in vitro (Wang et al., 2015; Yamazaki et al., 2018), and that in vitro roughly half of RBPs, including PRC2 (Davidovich et al., 2015), bind RNA unspecifically (Jankowsky and Harris, 2015). In fact, it seems likely that much of the affinity for *Xist* repeat E reflects RNA structure-based determinants as (when PLD2 is present) we see no binding at all to highly ordered 18S rRNA (Anger et al., 2013; Rabl et al., 2011). In the case of *Xist* its repeat E element is largely unstructured in vitro, and based on changes in selective 2′-hydroxyl acylation analyzed by primer extension reactivity, appears to be a major protein-binding platform in vivo (Smola et al., 2016) that is molded by its interactions. One possibility is that its inherent flexibility allows it to bypass restrictions imposed by PLD2. RNA structure has already been implicated in a polyQ-driven phase separation (Langdon et al., 2018; Zhang et al., 2015), and RNAs with large unstructured regions reported to form extensive intermolecular RNA–RNA interactions that play a role in formation of condensates with a mesh-like morphology (Ma et al., 2020 *Preprint*).

The second theme to emerge is the contribution of CIZ1’s polyQ tract (PLD1) to the shape and size of CIZ1 assemblies. In vitro, PLD1 has a dramatic effect on the structure and stability of CIZ1 self-assemblies, driving the unstable aggregates that form in the absence of PLD1 towards a stable branched fibrillar network. It also overrides the solubilizing effect of RNA, to one that promotes network growth. However, its relationship with repeat E is not straightforward. While PLD1 clearly drives high affinity interaction with repeat E (and other RNAs), repeat E serves to dampen CIZ1 self-assembly in vitro, implying that PLD1-driven self-assembly and PLD1-driven interaction with repeat E may compete. It is important to note, however, that the scale of the networks formed in vitro far exceeds that which might form inside nuclei, and further, that despite the presence of both PLD1 and PLD2, N571 sequences are insufficient to assemble at Xi inside cells.

The third key point is that CIZ1’s interaction with RNA is multivalent, because a functionally independent interface is encoded in the C-terminal 275 amino acids. When measured alone, this is relatively low affinity, forms a single discrete complex with RNA, and has no apparent preference for repeat E over repeat A (although it does prefer *Xist* over *Gapdh*). Recent studies have shown that in RBPs with well-ordered RNA-binding domains, their affinity for specific sequences can be driven by intrinsically disordered regions in the same protein (Corley et al., 2020), an effect also seen with DNA-binding specificity in vivo (Brodsky et al., 2020). Thus, the reported properties of PLD1 and PLD2 could confer greater affinity on C275. In fact, our data do show that N571 confers the ability to accumulate at Xi on C275 because, while neither domain alone is sufficient, together they form a single macromolecular assembly in female cells. Moreover, the involvement of two independent RNA interactions could confer a cross-linking effect that supports the formation of a network.

### Model

Together our interpretations of the data suggest a model in which multivalent interaction with RNA, coupled with PLD-driven self-association, support the formation of an RNA–CIZ1 matrix, localized to Xi by affinity for *Xist* repeat E (Fig. 6 F). We incorporate information on scaffold attachment factor A (SAF-A) which, like CIZ1, supports retention of *Xist* at Xi. SAF-A interacts directly with AT-rich scaffold/matrix attachment region (S/MAR) DNA across the genome via its serum amyloid P domain (Gohring et al., 1997; Kipp et al., 2000), and with *Xist* via its RGG domain (Helbig and Fackelmayer, 2003), through which it is proposed to form a bridge between RNA and DNA (Hasegawa et al., 2010). This would remain unaffected by the presence or absence of CIZ1, but be insufficient to maintain enrichment of *Xist* at Xi in differentiated cells to the extent that an *Xist* cloud is detected by FISH. Indeed, *Xist* is spread across the nucleus in the absence of CIZ1 (Ridings-Figueroa et al., 2017), demonstrating that SAF-A alone has limited capacity to retain *Xist* in differentiated cells. Retention of additional molecules of *Xist* would be dependent on CIZ1, captured initially via affinity for *Xist* repeat E, but augmented by secondary interactions with other RNAs to form a CIZ1–RNA matrix around Xi chromatin, that could serve to include or exclude other factors. Our data also indicate that interactions mediated by poly-glutamine domains are a determinant in CIZ1-*Xist* assembly shape, although we cannot conclude that this is mediated by PLD1 and could in fact be indirect.

*Xist* interactors, identified in proteomic and genetic screens (Chu et al., 2015; McHugh et al., 2015; Moindrot et al., 2015; Monfort et al., 2015), are enriched in glutamine-rich RBPs with a high probability of phase separating (Cerase et al., 2019), the potential of which has already been hypothesized to play a role in the formation of the repressive Xi chromatin compartment. Supporting evidence for this has emerged from analysis of the PTBP1, MATR3, TDP-43, and CELF1 *Xist*-dependent protein assembly during embryonic stem cell differentiation. Assembly formation is also driven by interaction with *Xist* repeat E during the later stages of initiation of X-inactivation, and it serves the dual function of being required for gene silencing as well as anchoring *Xist* to the Xi territory (Pandya-Jones et al., 2020). This analysis defines a time window when repeat E is essential, but suggests that in cells, CIZ1 is recruited to Xi via repeat E independently of these factors (Pandya-Jones et al., 2020). Our own analysis suggests that CIZ1 is recruited during the initiation of silencing, but that it is not required for its establishment (Ridings-Figueroa et al., 2017). However, none of the preceding data consider the confounding effect of CIZ1 alternative splicing, which may well determine when CIZ1 is competent to be captured by repeat E. Together, the available data argue that CIZ1’s function is in the later, maintenance phase of X inactivation, where it is required for high-fidelity maintenance of polycomb-regulated genes (Stewart et al., 2019).

### Alternative splicing

Extensive CIZ1 splice variant diversity exists in both somatic and germline cells in the mouse (Greaves et al., 2012), but CIZ1 alternative splicing is even more complex in humans (Rahman et al., 2007; Rahman et al., 2010). The first CIZ1 variant implicated in human disease, the pediatric central nervous system tumor medulloblastoma, lacks PLD1 (Warder and Keherly, 2003); more recently, exome sequencing has revealed polymorphisms in the length of the polyQ tract in PLD1, with deletion of nine glutamines reported in seven malignant tumors of different origins. A range of other single nucleotide polymorphisms, evident in both PLD1 and PLD2, are also listed in Catalogue of Somatic Mutations in Cancer (Tate et al., 2019; Table S1), and further changes in CIZ1 are linked with both benign (Wang et al., 2019) and malignant (Chen et al., 2020; Liu et al., 2015; Wang et al., 2014; Wu et al., 2016; Zhang et al., 2014) tumors. CIZ1-associated pathologies are not limited to cancer, however. Alterations that impact PLD1, or that occur elsewhere and influence alternative splicing and tendency to aggregate inside cells, are also linked with familial cervical dystonia (Xiao et al., 2012; Xiao et al., 2014) and Alzheimer’s disease (Dahmcke et al., 2008), and other mutations with benign essential blepharospasm (Dong et al., 2019). Thus, modulation of CIZ1 nuclear assembly formation may play a role in diverse human pathologies, most of which have yet to be fully explored in relation to maintenance of epigenetic state.

### Phase separation

Despite clear evidence for dependence on PLD1 for both in vitro and in vivo assemblies, we cannot conclude that CIZ1–Xi assemblies form by LLPS or that they are fluid. Complicating this question is the observation that the stability (and possible fluidity) of CIZ1 assemblies fluctuates in the cell cycle, as revealed by temporally resolved subnuclear fractionation (Stewart et al., 2019). This shows that CIZ1–Xi assemblies are independent of DNA because they remain unperturbed by removal of chromatin (identifying them as part of a classic “nuclear matrix”), but are dispersed upon digestion of RNA during most of the cell cycle. Importantly, for a brief window in S phase, during or immediately after Xi chromatin replication, CIZ1–Xi assemblies become resistant to removal of RNA as well as DNA, defining a transitional state that coincides with a shift in Xi location and maintenance of gene silencing (Stewart et al., 2019; Zhang et al., 2007). The molecular mechanisms that underlie this transient change in CIZ1 assembly cohesion now deserve further attention.

In summary, the data presented here demonstrate the contribution of two polyQ-rich domains to the formation of *Xist*-dependent CIZ1 assemblies at Xi. Because CIZ1 was previously shown to stabilize polycomb target gene expression at Xi and elsewhere (Stewart et al., 2019), these findings implicate a polyQ domain in the maintenance of the epigenetic state.

## Materials and methods

### Mouse cell lines and culture

CIZ1-null mice were generated from C57BL/6 ES clone IST13830B6 (TIGM) harboring a neomycin resistance gene trap inserted downstream of exon 1. The absence of *Ciz1*/CIZ1 in homozygous progeny was confirmed by quantitative PCR, immunofluorescence, and immunoblot. Mouse PEFs were derived from day 13 or 14 embryos and genotyped as described (Ridings-Figueroa et al., 2017). They were cultured in DMEM containing 10% FCS (PAAgold), 100 units/ml penicillin, 10 µg/ml streptomycin, and 2 mM L-glutamine up to a maximum of passage 4. After passage 4, these cells are referred to as MEFs, which were not used here. For inducible cells harboring transactivator and responder transgenes, addition of doxycycline to medium (10 μg/ml) was used to induce GFP-CIZ1 for 24–48 h as indicated. Female 3T3 cells D001 were grown in DMEM (Sigma-Aldrich), 1% penicillin, streptomycin, glutamine (Gibco), and 10% FBS (Stewart et al., 2019). The amidosulfonamide inhibitor of polyQ aggregation C2-8 (*N*-(4-bromophenyl)-3-[[(4-bromophenyl)amino]sulfonyl]benzamide; Sigma-Aldrich) was prepared at 10 mM in DMSO and used at the indicated concentrations in cell culture media for 24 or 48 h as indicated.

### Mammalian expression constructs and transfection

Murine GFP-CIZ1 (full length, 845 amino acids) and GFP-CIZ1Δ2p6p8 (splice variant formerly known as ECIZ1) are expressed in pEGFP-C3 (Clontech; Coverley et al., 2005). Derived N-terminal fragments N571 and N442 were generated by restriction digestion (Ainscough et al., 2007). GFP-CIZ1 C275 was made by ligating the 1-kb C-terminal XhoI fragment (Coverley et al., 2005) into the XhoI site of pEGFP-C2 (Clontech). GFP-CIZ1Δp8 (ΔPLD2, this study) was made by replacing the p8 containing BcuI/PmlI fragment of GFP-CIZ1(845) with the Δp8 BcuI/PmlI fragment of GFP-CIZ1Δ2p6p8. GFP-CIZ1Δ2p6 was made by replacing the Δp8 BcuI/PmlI fragment of GFP-CIZ1Δ2p6p8 with the p8 containing BcuI/PmlI fragment of GFP-CIZ1(845). GFP-CIZ1ΔE2 (ΔPLD1, this study) was made by replacing the Δp6 KflI/PmlI fragment of GFP-CIZ1 Δ2p6 with the p6 containing KflI/PmlI fragment of GFP-CIZ1(845). GFP-CIZ1Δp6 was generated by deleting residues (Δ197–201) using site-directed mutagenesis. Constructs were introduced into PEFs or 3T3 cells using Mirus X2 transfection and analyzed after 24 or 48 h.

### Protein expression and purification

All recombinant proteins were expressed and purified from the bacterium *Escherichia coli* strain BL21-CodonPlus-RP. Cells were grown in Luria broth (starter culture) and routinely cultured at 37°C while shaking at 220 rpm. Mouse CIZ1Δ2p6p8, C275, C181, N571, N571ΔPLD1 (Δ0–67 aa), N571ΔPLD2 (Δ361–399 aa), and N442 in frame with N-terminal tag glutathione S-transferase (GST) in pGEX-6P-3 expression plasmids (GE Healthcare) were expressed in BL21-CodonPlus-RP *Escherichia coli* using lactose-driven autoinduction at 20°C. Cells were harvested after 24 h at 20°C to produce 6–7 g of bacterial cell paste and frozen. Pellets were thawed on ice, suspended in 30–35 ml cold Hepes-buffered saline (50 mM Hepes, pH 7.8 at 25°C, 135 mM NaCl, 3 mM EDTA, and 1 mM DTT), supplemented with EDTA-free EZBlock protease inhibitor cocktail (BioVision) and 1 mM PMSF. Cells were sonicated on ice for five cycles (15 s on, 30 s off) at 60% amplitude using a 6-mm probe (microtip MS 73; Bandelin SONOPULS). Lysates were clarified by centrifugation for 20 min 4°C at 15,000 rpm in a Heraeus Multifuge X1 centrifuge with F15-6x100y fixed angle rotor. For affinity purification, all steps were performed at 4°C. Clarified lysates were incubated with prewashed glutathione Sepharose (GE Healthcare), gently mixed with rotation for 1 h at 4°C, and transferred to an equilibrated Poly-Prep chromatography column, 0.8 × 4 cm (Bio-Rad). For removal of nucleic acids and unbound proteins, the column was washed with 10 column volumes (c.v.) of cold wash buffer 1 (50 mM Hepes, pH 7.8 at 25°C, 1 M NaCl, 3 mM EDTA, and 1 mM DTT), followed by three washes with 10 c.v. cold wash buffer 2 (50 mM Hepes, pH 7.8 at 25°C, 135 mM NaCl, 3 mM EDTA, and 1 mM DTT). Bead-bound protein was gradually eluted with 4 × 2 c.v. of elution buffer (50 mM Tris-HCl, pH 8.0 at 25°C, and 10 mM L-glutathione reduced) by agitating beads for 10 min at 4°C. Identity and purity of CIZ1-containing elution fractions was assessed by SDS-PAGE with SimplyBlue safe stain (Invitrogen) and prestained protein ladder 10–250 kD (Thermo Fisher Scientific). CIZ1-containing fractions were pooled, and reduced glutathione was removed by buffer exchange (50 mM Tris-HCl, pH 7.0 at 25°C, 150 mM NaCl, 1 mM EDTA, and 1 mM DTT) using Zeba Desalt spin columns (Thermo Fisher Scientific) following the manufacturer’s instructions. GST tag was removed by incubating with 2 units of PreScission protease (GE Healthcare) per 100 µg protein for 16–18 h at 4°C. Cleavage efficiency and specificity were examined by running an appropriate volume of digestion mixture on SDS-PAGE. Digestion mixture was passed over fresh glutathione Sepharose, and pure CIZ1 fractions were collected and concentrated with a Vivaspin concentrator, 10-kD cutoff (GE Healthcare), or a Pierce concentrator, 20-kD cutoff (Thermo Fisher Scientific). Protein concentration was determined by absorbance at 280 nm with NanoDrop ND-1000 spectrophotometer (v3.2.1; Labtech). Absence of nucleic acids was verified by ensuring that the ratio of UV absorbance at 260–280 nm was ≤0.7 and by visualization using denaturing gel electrophoresis. Immunoblot verification of purified CIZ1 proteins typically used 2 µg purified protein per lane, detected with antibodies listed in Table S3. Purified proteins were supplemented to 9% vol/vol (final concentration) with sterile glycerol, aliquoted, and snap frozen in nuclease-free cryotubes (Nunc) in liquid nitrogen and stored at −80°C.

### In vitro transcription of digoxigenin (DIG)-labeled probes

DNA templates used for in vitro transcription of mouse *Xist* RNA were amplified by PCR from sequence-verified plasmid pCMV-Xist-PA (Wutz and Jaenisch, 2000; 26760; Addgene), containing the murine *Xist* gene, using high-fidelity Platinum *pfx* DNA polymerase (Invitrogen). PCR primers used for the amplifications contained T7 promoter sequences, designed with SnapGene (GraphPad software), and are shown in Table S2. PCR amplicons of predicted size were confirmed by agarose gel electrophoresis with DNA ladders prior to in vitro transcription and afterward purified using QIAquick Gel Extraction Kit (Qiagen) for sequence verification. To generate human 18S rRNA probe, as an example of a highly structured template (Anger et al., 2013), the pTRI-RNA 18S control construct (MEGAshortscript T7 Transcription Kit; Ambion) was used as a DNA template for the in vitro transcription, to produce a 128-nucleotide product. To generate mouse *gapdh* RNA probe, the *pTRI-GAPDH* control construct (NorthernMax-Gly kit; Ambion) was used as a DNA template for in vitro transcription to produce a 387-nucleotide product. In vitro transcription reactions were carried out in 0.2-ml thin-walled tubes with T7 RNA polymerase (MEGAshortscript T7 Transcription Kit; Ambion) at 37°C for 4 h. Upon completion, reactions were incubated with appropriate amount of RNase-free TURBO DNase (Ambion) at 37°C for 15–25 min to digest template DNA, and later RNA transcripts were purified with MEGAclear Kit (Ambion) following the manufacturer’s instructions. RNAs were eluted with elution buffer (nuclease-free water and 0.1 mM EDTA, pH 8.0) and quantified by UV absorbance at 260 nm with a NanoDrop ND-1000 spectrophotometer (v3.2.1; Labtech). The RNA samples were mixed with gel loading buffer II (95% formamide, 18 mM EDTA, 0.025% SDS, Xylene Cyanol, and Bromophenol Blue) at a 1:1 ratio, incubated for 3 min at 80°C, and loaded while still hot on denaturing gels (5% wt/vol 29:1 acrylamide/bisacrylamide with 7 M urea) buffered with 1× Tris/Borate/EDTA, and run at 130 V. Purity, transcript size, and integrity of all RNA constructs were examined with denaturing PAGE gels stained with SYBR Safe DNA Gel Stain (Invitrogen), with RNA Century-Plus Markers 0.1–1 kb (Ambion). All in vitro–transcribed RNA transcripts were labeled by incorporating digoxigenin-11-UTP (DIG) at a ratio of 1:15 with unmodified UTP during in vitro transcription.

### EMSA

In a 10-μl binding reaction, purified recombinant CIZ1 proteins and derived fragments (CIZ1Δ2p6p8, C275, C181, N571, N571ΔPLD1, N571ΔPLD2, and N442) were incubated with DIG-labeled RNA probes in binding buffer (10 mM Tris-HCl, pH 7.5 at 25°C, 30 mM NaCl, 2.5 mM MgCl_2_, 0.2 mM DTT, 0.1% IGEPAL CA-630 [Fluka], 0.1 mg/ml yeast tRNA [Ambion], 0.4 units RNaseOUT [Invitrogen], and 1% vol/vol glycerol) at 30°C for 20 min. Before use RNA was denatured at 80°C for 3 min and snap cooled on ice for 2–3 min to allow RNA refolding. Reaction mixtures were loaded onto an 11 × 6-cm horizontal nondenaturing 0.7% agarose gel (Molecular Biology Grade agarose) buffered with 1× filter-sterilized Tris/Borate/EDTA at 4°C. Gel electrophoresis was carried out for 60 min at 6 V/cm in an icebox in a 4°C cold room. Blotting was performed at room temperature by upward capillary transfer onto a positively charged nylon membrane (Hybond-N+) for 45 min. The membrane was placed on blotting paper equilibrated with 2× SSC buffer, and the membrane and RNA were cross-linked by exposure to shortwave UV light at 254 nm and 120 mJ (GS Gene linker UV Chamber; Bio-Rad). Membranes were washed in 1× wash buffer (0.1 M maleic acid, 0.15 M NaCl, pH 7.5 at 20°C, and 0.3% [vol/vol] Tween-20) for 5 min under agitation and next blocked in 1× blocking buffer (Roche) for 30 min at room temperature with gentle shaking. The membrane was incubated with a polyclonal sheep anti-digoxigenin antibody conjugated to alkaline phosphatase (Roche) at 1:10,000 dilution for 30 min with gentle shaking, then washed twice in 1× wash buffer at room temperature for 15 min with gentle shaking and equilibrated in 1× detection buffer (100 mM Tris-HCl, pH 9.5, and 100 mM NaCl) for 5 min at room temperature. The membrane was developed by adding chemiluminescent chloro-5-substituted adamantyl-1,2-dioxetane phosphate substrate (Roche) at 0.25 mM final concentration at room temperature for 5 min and membrane incubated at 37°C for 10 min for alkaline phosphatase activation. The chemiluminescent signal was acquired with a PXi touch Chemiluminescence imaging system (Syngene). Densitometry was carried out with GeneTools software (v4.3.8.0; Syngene). All quantifications were expressed relative to the lane containing RNA probe but no protein for each gel, and results expressed as percentage, averaged across three independent replicate experiments. Error bars show SEM.

### In vitro assemblies

Purified proteins in 50 mM Tris-HCl, pH 7.0, 150 mM NaCl, and 1 mM DTT were concentrated to greater than 60 µM stock solution and diluted in isolation buffer as appropriate. The reactions (10 μl final volume) were assembled in 0.2-ml tubes and mixed by pipetting, and incubations were carried out in uncoated multiwell glass-bottom plates at room temperature, unless indicated otherwise. Total cellular RNA was isolated from a female 3T3 cell line (Ridings-Figueroa et al., 2017) using Trizol reagent as recommended. Transfer RNA was from yeast (Ambion). Samples were imaged using an Evos Xl (AMG) light microscope fitted with an Evos fluorite LWD phase-contrast 40× 0.65 objective, using constant illumination settings and capture times within a sample series to generate 2,048 × 1,536–pixel, 9.4 MB TIFF image files, where 1 pixel is 0.226 µm. Image sets were processed using Fiji software to produce optimized contrast for reproduction purposes. Quantitative analysis of skeletons and particles was performed on nonadjusted images. For particle size (in pixels), particle number, and circularity measurements, 250 × 250–pixel image sections were converted to binary output using Fiji, and all particles (0 to infinity) were analyzed. Circularity reflects the smoothness of the perimeter of an object, where a perfect circle has a circularity value of 1.0, using the formula 4π(area/perimeter^2^). For branch analysis, binary images were converted to skeletons and analyzed without pruning. At least five images were analyzed for each condition, and results were summarized to yield mean data for each parameter, including branch termini (end-point voxels), and branch length (maximum branch length per particle expressed as average per field), or nonjunction, nonterminus skeleton body (slab voxels), referred to as skeleton size. Note, that highly assembled particles (e.g., late time points for N571) grew in X, Y, and Z, creating high-contrast phase images for which skeletonization revealed gaps between assemblies rather than assemblies themselves, necessitating image inversion. Typically, sample sets were compared by Student’s two tailed *t* test in Excel. Statistical symbols: *, P < 0.05; **, P < 0.01; and ***, P < 0.001.

### RNA FISH

Fluorescently labeled RNA FISH probe was produced from mouse pCMV-*Xist-*PA plasmid harboring 15 kb *Xist* insert (Wutz and Jaenisch, 2000, plasmid, 26760; Addgene) and purified using GENECLEAN kit (MPBIO). An 11-kb Spe1-Sal1 fragment (mid exon 1–7) was labeled with either Cy3-dUTP (PA53022; GE Healthcare UK) or ChromaTide Alexa Fluor 594-5-dUTP (C11400; Invitrogen) using Bioprime DNA labelling kit (18094-011; Invitrogen). 100 ng *Xist* fragment was added to 20 μl 2.5× random primer buffer and 9 μl nuclease-free water, denatured by boiling for 5 min, and incubated on ice for 2 min. dNTPs (5 μl dATP, dCTP, and dGTP; 3 μl dTTP; and 1 μl labeled dUTP) were added under low light, then 1 μl Klenow fragment, and incubated overnight at 37°C in the dark. After incubation, 5 μl stop buffer, 10 μl Cot1 DNA (Invitrogen) and 5 μl salmon sperm DNA (Novagen) were added. Labeled DNA was precipitated twice with 3 M NaOAc and 100% ethanol to remove unincorporated nucleotides, resuspended in 80 μl hybridization buffer (50% formamide, 10% dextran sulfate, 2 mg/ml BSA, and 2× SSC), and stored at −20°C. Cells were seeded onto glass coverslips at ∼70% density and transfected as required. After 24 h, cells were washed in RNase free PBS and fixed in fresh 4% PFA, on ice, for 15 min. Cells were washed in PBS (3× 5 min) and incubated in 1 ml permeabilization solution (PBS + 0.5% Triton X-100 [Sigma-Aldrich], 0.5% BSA [Jackson ImmunoResearch]) with 10 mM vanadyl ribonucleoside complex (NEB) per coverslip for 10 min at room temperature. The cells were washed in PBS (3 × 5 min) and stored at 4°C in 70% ethanol. Labeled probe (10 μl per coverslip) was defrosted on ice, and vanadyl ribonucleoside complex was added to 10 mM. The probe was denatured (74°C, 10 min), spun briefly, and incubated at 37°C for 20 min. Prepared cells were dehydrated through an alcohol series of 70%, 80%, 95%, and 100% ethanol for 2 min each. Coverslips were air dried for 5 min, placed cell side down onto a 10-μl denatured probe on an RNase free slide, sealed with rubber cement, and incubated at 37°C overnight in a humidified chamber in the dark. Coverslips were carefully lifted and sequentially washed with 2× SSC, 50% formamide (3× 5 min, 39°C), and 2× SSC (3× 5 min, 39°C), and then once each in 1× SSC and 4× SSC for 5 min at room temperature. After a brief dip in diethyl pyrocarbonate water, coverslips were mounted in Vectashield with DAPI (H-1200; Vector Labs) or processed for immuno-FISH.

### Immunofluorescence

For immunofluorescence, cells were grown on coverslips, washed in PBS, and fixed in 4% PFA to reveal total protein, or alternatively, briefly washed in PBS with 0.1% Triton X-100 then fixed in 4% PFA, to reveal the immobilized protein fraction (detergent treated). For combined RNA/immuno-FISH, cells were processed for RNA as described then continued as below. Coverslips were blocked in AB (1× PBS, 10 mg/ml BSA, 0.02% SDS, and 0.1% Triton X-100) for 30 min, incubated with primary antibodies for 2 h at 37°C, washed in AB, incubated with secondary antibodies for 1 h at 37°C, washed three times, and mounted on glass slides with Vectashield medium containing DAPI (Vector Labs). In the indicated experiments, cells were mounted in Vectashield medium without DAPI, and instead DAPI (0.5 μg/ml) was included in the final wash step. All antibodies used are detailed in Table S3. Alexa Fluor 568 (red) or 488 (green) was used for detection in all cases. Fluorescence images were captured using a Zeiss Axiovert 200M fitted with a 63×/1.40 Plan-Apochromat objective and Zeiss filter sets 2, 10, and 15 (G365 FT395 LP420, BP450-490 FT510 BP515-565, and BP546/12 FT580 LP590), using Axiocam 506 mono and Axiovision image acquisition software (SE64 release 4.9.1) through Zeiss Immersol 518F. Where fluorescence intensity is quantified, cells were imaged as a set, with all images for each filter set captured with the same exposure time at 21°C (room temperature). Images were saved at 1,499 × 1,205 pixels in tagged image file format for downstream analysis. Image quantification was done on unmodified images. For area measures, region masks were generated in blue (nucleus) and green (CIZ1) or red (*Xist*) using Fiji Otsu threshold (particles between 0.1 inches and infinity were selected including holes), and area occupied by CIZ1 or *Xist* assemblies expressed as proportion of nuclear area. For frequency scores, typically analysis was carried out directly on samples. Experiments were designed to use the minimum number of animal-derived primary cell populations while achieving statistically valid data in two or more independent experiments. For single-parameter endpoints such as analysis of the frequency of CIZ1 assemblies, where the variable is a mutation across a set of sampling times, the output from typically three biological replicates (independent PEF lines) is compared by two tail Student’s *t* test in Excel. For 3T3 cell experiments, replicate numbers were typically far in excess of three and are stated in the legends. In all cases, data distribution was assumed to be normal, but this was not formally tested. Frequencies at each data point are scored by eye, and avoidance of bias was achieved by independent, blinded analysis of archived images. Unless indicated, data are represented as means, error bars show SEM, and data were compared by Student’s two-tailed *t* test. For reproduction purposes, images were enhanced, split, or cut using Fiji, in all cases to accurately reflect actual relationships between factors that were quantified from unmodified images. A list of project tools and reagents is given in Table S4.

### Ethics

All work with animal models is compliant with UK ethical regulations. Breeding of mice was carried out under UK Home Office license and with the approval of the Animal Welfare and Ethical Review Body at the University of York and Oxford. Analysis on cells and tissues derived from these mice was carried out with the approval of the Animal Welfare and Ethical Review Body at the University of York.

### Bioinformatics

Protein motifs were identified using Psort II (https://psort.hgc.jp/). Alignments were performed using Clustal omega. Protein disorder was predicted using MobiDB (https://mobidb.bio.unipd.it/), PONDR (http://www.pondr.com/), and disEMBL (http://dis.embl.de/), and PLD using PLAAC (http://plaac.wi.mit.edu/).

### Online supplemental material

Fig. S1 shows Xi accumulation of GFP-CIZ1 and derived mutants and its sequence requirements for nuclear localization. Related to Fig. 1. Fig. S2 shows comparison between mouse and human CIZ1 depicting similarity in alternative splicing, PLDs and structural disorder predictions. Related to Fig. 1. Fig. S3 shows de novo formation of ectopic GFP-CIZ1 assemblies at Xi and recruitment of *Xist* and repressive marks. It also shows the effect of C2-8 on the shape of CIZ1 and *Xist* assemblies. Related to Figs. 2 and 3. Fig. S4 shows additional in vitro assembly data and controls for N571 and PLD deletion mutant. Related to Figs. 3 and 4. Fig. S5 shows additional EMSA data and controls for N- and C-terminal fragments of CIZ1. Related to Figs. 5 and 6. Table S1 shows sequence variations in the PLD domains of human CIZ1. Table S2 lists primers used for making DNA templates for in vitro transcription. Table S3 lists antibodies used for immunofluorescence and immunoblotting. Table S4 lists project tools and reagents.

## Supplementary Material

Table S1 lists the summary of reported pathology-associated sequence variations in human CIZ1 PLD domains, including those documented more than once in COSMIC human tumor samples (Tate et al., 2019).

Table S2 lists the primers used to generate DNA templates for in vitro transcription.

Table S3 lists the antibodies used for immunofluorescence and Western blot studies.

Table S4 provides the list of other reagents and specific tools.
