# Supplementary material for: Prion-like domains drive CIZ1 assembly formation at the inactive X chromosome
Source: J Cell Biol. 2022 Mar 15;221(4):e202103185. doi: 10.1083/jcb.202103185 (PMC8927971; doi:10.1083/jcb.202103185)
Supplement: Table S1 — lists the summary of reported pathology-associated sequence variations in human CIZ1 PLD domains, including those documented more than once in COSMIC human tumor samples (Tate et al., 2019). [file JCB_202103185_TableS1.docx]

**Supplemental Table 1**

Summary of reported pathology-associated sequence variations in human CIZ1 PLD domains, including those documented more than once in COSMIC human tumour samples (Tate et al., 2019)**.**

| **Sequence variation** | **Disease** | **Source** |
| --- | --- | --- |
| In frame deletion of Q9 in PLD1 | Adrenal gland Malignant Pheochromocytoma  Large intestine Adenocarcinoma  Large intestine Adenocarcinoma  Liver Carcinoma  Malignant melanoma  Malignant melanoma  Brain Haemangioblastoma | GRCh38·COSMIC v90 |
| SNP in PLD1 (exon 2) generating L36P | Thyroid Carcinoma | GRCh38·COSMIC v90 |
| SNP in PLD1 (exon 2) generating P47S | Cervical dystonia | (Xiao et al., 2012) |
| SNP in PLD1 (exon 2) generating R57W | Tongue Squamous cell carcinoma  Tongue Squamous cell carcinoma | GRCh38·COSMIC v90 |
| SNP in PLD1 (exon 2) generating L59P | Astrocytoma Grade IV  Astrocytoma Grade IV  Glioma  renal cell carcinoma | GRCh38·COSMIC v90 |
| Exclusion of PLD 1 (exons 2, 4, 6) | Medulloblastoma | (Warder and Keherly, 2003) |
| SNP in (exon 7) S264G with effects on splicing and nuclear aggregate formation | Cervical dystonia | (Xiao et al., 2012) |
| SNP in PLD2 (exon 8) generating Q360* | Lung Adenocarcinoma  Stomach Adenocarcinoma | GRCh38·COSMIC v90 |
| Conditional exclusion of part of PLD2 (part exon 8) | Alzheimer’s disease | (Dahmcke et al., 2008) |
