# Supplementary material for: Prion-like domains drive CIZ1 assembly formation at the inactive X chromosome
Source: J Cell Biol. 2022 Mar 15;221(4):e202103185. doi: 10.1083/jcb.202103185 (PMC8927971; doi:10.1083/jcb.202103185)
Supplement: Table S2 — lists the primers used to generate DNA templates for in vitro transcription. [file JCB_202103185_TableS2.docx]

**Supplemental Table 2**

Primers used to generate DNA templates for *in vitro* transcription.

| **Probe** | **Length** | **Forward** | **Reverse** |
| --- | --- | --- | --- |
| RepA sense | 1-500 | T7-F: TAATACGACTCACTATAGGGAGCTTGCTCCAGCCATGTTTGCTCG | R: CTAAGGAGAAGAAAAAAAGAATAAAAGC |
| RepE proximal | 1-474 | T7-F: TAATACGACTCACTATAGGGAGATTTCTTCCTTGCAGTTGTGTCTAATTC | R: ACAGAGAGCCATAGCTAGTGAAG |
| RepE distal sense (20 nucleotide overlap with RepE proximal) | 1-472 | T7-F: TAATACGACTCACTATAGGGAGCACTAGCTATGGCTCTCTG | R: CACATAACACACATGCACACACGC |
| RepE distal antisense | 1-472 | F: CACTAGCTATGGCTCTCTGTTTTATCTATCTG | T7-R: TAATACGACTCACTATAGGGAGCACATAACACACATGCACACACGC |
