# Supplementary material for: Prion-like domains drive CIZ1 assembly formation at the inactive X chromosome
Source: J Cell Biol. 2022 Mar 15;221(4):e202103185. doi: 10.1083/jcb.202103185 (PMC8927971; doi:10.1083/jcb.202103185)
Supplement: Table S3 — lists the antibodies used for immunofluorescence and Western blot studies. [file JCB_202103185_TableS3.docx]

**Supplemental Table 3**

Antibodies used for immunofluorescence and western blot studies.

| **Antibodies** | **Source** |
| --- | --- |
| 1794 (N-term CIZ1) | Coverley et al., 2005 |
| C- term mCIZ1 | Novus (NB100-74624) |
| C-term CIZ1 | Biorbyt (329770) |
| C- term hC221a | (Stewart et al., 2019) |
| α Mouse H3K27me3 | Abcam (6002) |
| Rabbit H3K27me3 | CST (9733S) |
| Rabbit H2AK119Ub | CST (8240) |
| lamin B | Invitrogen 33-2100 |
| Rabbit anti-GST | Abcam ab9085 |
| Goat α Rabbit Alexa Fluor 568 (Red) | Invitrogen (A11011) |
| Goat α Rabbit Alexa Fluor 488 (Green) | Invitrogen (A11034) |
| Goat α Mouse Alexa Fluor 568 (Red) | Invitrogen (A11031) |
| Goat α Mouse Alexa Fluor 488 (Green) | Invitrogen (A11001) |
| Peroxidase IgG α Rabbit | Jackson 211-032-171 |
| Peroxidase IgG α Mouse | Jackson 155-035-174 |
| Sheep polyclonal anti-digoxigenin-AP | Roche 11093274910 |
