# Supplementary material for: Prion-like domains drive CIZ1 assembly formation at the inactive X chromosome
Source: J Cell Biol. 2022 Mar 15;221(4):e202103185. doi: 10.1083/jcb.202103185 (PMC8927971; doi:10.1083/jcb.202103185)
Supplement: Table S4 — provides the list of other reagents and specific tools. [file JCB_202103185_TableS4.docx]

**Supplemental Table 4**

List of other reagents and specific tools

| REAGENT or RESOURCE | SOURCE | IDENTIFIER |
| --- | --- | --- |
| Bacterial and Virus Strains | | |
| *Escherichia coli* BL21-Codon plus-RP | Agilent Technologies | Cat#230250 |
| Biological Samples | | |
| Chemicals, Peptides, and Recombinant Proteins | | |
| Chemiluminescent CSPD | Roche | Cat#11655884001 |
| TURBO DNase | Ambion | Cat#AM1354 |
| Protease Inhibitor Cocktail, EDTA-Free | BioVision | Cat#K272-1 |
| PreScission protease | GE Healthcare | Cat#27084301 |
| Platinum™ *Pfx* DNA Polymerase | Invitrogen | Cat#11708021 |
| RNase OUT™ | Invitrogen | Cat#10777019 |
| IGEPAL® CA-630 | Fluka | Cat#56741 |
| Recombinant GST- CIZ1Δ2p6p8 (ECIZ1) | Copeland et al., 2015 | N/A |
| Recombinant truncated GST-N571 | Coverley et al., 2005 | N/A |
| Recombinant truncated GST-N442 | Coverley et al., 2005 | N/A |
| Recombinant truncated C275 | Coverley et al., 2005 | N/A |
| Recombinant truncated C181 | This study | N/A |
| Recombinant truncated GST-N571ΔPLD1 | This study | N/A |
| Recombinant truncated GST-N571ΔPLD2 | This study | N/A |
| Critical Commercial Assays | | |
| MEGAshortscript™ T7 Transcription Kit | Ambion | Cat#AM1354 |
| MEGAclear™ Transcription Clean-Up Kit | Ambion | Cat#AM1908 |
| QIAquick Gel Extraction Kit | QIAGEN | Cat#28704 |
| GENECLEAN® kit | MPBIO | Cat#111102-200 |
| BioPrime™ labelling kit | Invitrogen | Cat#18094-011 |
| Deposited Data | | |
| Experimental Models: Cell Lines | | |
| Female 3T3 cells D001 | Stewart et al., 2019 | N/A |
| Mouse PEFs | Ridings-Figueroa et al., 2017 | N/A |
| Experimental Models: Organisms/Strains | | |
| Ciz1 null mice (IST13830B6)TIGM | Ridings-Figueroa et al., 2017 | N/A |
| Oligonucleotides |  |  |
| List and sequences of primers (Supplemental Table 2) | This manuscript | N/A |
| Recombinant DNA | | |
| Mouse pCMV-Xist-PA | Wutz et al., 2000 | Addgene plasmid # 26760 |
| Murine GFP-CIZ1 | Coverley et al., 2005 | N/A |
| Murine GFP- CIZ1Δ2p6p8 (ECIZ1) | Coverley et al., 2005 | N/A |
| Murine GFP- CIZ1Δp8 (ΔPLD2) | This paper | N/A |
| Murine GFP- CIZ1Δp6 | This paper | N/A |
| Murine GFP- CIZ1Δ2 (ΔPLD1) | This paper | N/A |
| Murine GFP- C275 | Ainscough et al., 2007 | N/A |
| Murine GFP- N571 | Ainscough et al., 2007 | N/A |
| Murine GFP- N442 | Ainscough et al., 2007 | N/A |
| Software and Algorithms | | |
| GeneTools analysis software, version 4.3.8.0 | Syngene | https://www.syngene.com/software/genetools-automatic-image-analysis/ |
| PLAAC | Lancaster et al., 2014 | <http://plaac.wi.mit.edu/> |
| PONDR |  | <http://www.pondr.com/> |
| MobDB | Piovesan et al., 2021 | https://mobidb.bio.unipd.it/) |
| Conserved Domain Database |  | <https://www.ncbi.nlm.nih.gov/Structure/cdd/wrpsb.cgi>) |
| Fiji - ImageJ |  | <https://imagej.net/Fiji> |
| EVOS™ Xl (AMG) light microscope |  | N/A |
| Zeiss Axiovert 200M inverted microscope |  | N/A |
| Other | | |
| Digoxigenin-11-UTP | Roche | Cat#11209256910 |
| DIG Wash and Block Buffer Set | Roche | Cat#11585762001 |
| Yeast tRNA | Ambion | Cat#AM7119 |
